# In vitro oxidative decarboxylation of free fatty acids to terminal alkenes by two new P450 peroxygenases

**DOI:** 10.1186/s13068-017-0894-x

**Published:** 2017-09-07

**Authors:** Huifang Xu, Linlin Ning, Wenxia Yang, Bo Fang, Cong Wang, Yun Wang, Jian Xu, Severine Collin, Frederic Laeuffer, Laurent Fourage, Shengying Li

**Affiliations:** 1 Shandong Provincial Key Laboratory of Synthetic Biology, Qingdao Institute of Bioenergy and Bioprocess Technology, Chinese Academy of Sciences, No. 189 Songling Road, Qingdao, 266101 Shandong China; 2 CAS Key Laboratory of Biofuels, Qingdao Institute of Bioenergy and Bioprocess Technology, Chinese Academy of Sciences, No. 189 Songling Road, Qingdao, 266101 Shandong China; 3 Single-Cell Center, Qingdao Institute of Bioenergy and Bioprocess Technology, Chinese Academy of Sciences, No. 189 Songling Road, Qingdao, 266101 Shandong China; 4 University of Chinese Academy of Sciences, Beijing, 100049 China; 5 Total Refinery and Chemistry, SDR/Biofuels, Tour Coupole, 2, PI. Jean Millier, 92400 Courbevoie, France

**Keywords:** Alkenes, Biofuels, P450 fatty acid decarboxylases, Substrate specificity, Chemoselectivity, Site-directed mutagenesis, Enzyme kinetics

## Abstract

**Background:**

P450 fatty acid decarboxylases represented by the unusual CYP152 peroxygenase family member OleT_JE_ have been receiving great attention recently since these P450 enzymes are able to catalyze the simple and direct production of 1-alkenes for potential applications in biofuels and biomaterials. To gain more mechanistic insights, broader substrate spectra, and improved decarboxylative activities, it is demanded to discover and investigate more P450 fatty acid decarboxylases.

**Results:**

Here, we describe for the first time the expression, purification, and in vitro biochemical characterization of two new CYP152 peroxygenases, CYP-Aa162 and CYP-Sm46Δ29, that are capable of decarboxylating straight-chain saturated fatty acids. Both enzymes were found to catalyze the decarboxylation and hydroxylation of a broad range of free fatty acids (C_10_–C_20_) with overlapping substrate specificity, yet distinct chemoselectivity. CYP-Sm46Δ29 works primarily as a fatty (lauric) acid decarboxylase (66.1 ± 3.9% 1-undecene production) while CYP-Aa162 more as a fatty (lauric) acid hydroxylase (72.2 ± 0.9% hydroxy lauric acid production). Notably, the optical spectroscopic analysis of functional CYP-Sm46Δ29 revealed no characteristic P450 band, suggesting a unique heme coordination environment. Active-site mutagenesis analysis showed that substitution with the proposed key decarboxylation-modulating residues, His85 and Ile170, enhanced the decarboxylation activity of CYP-Aa162 and P450_BSβ_, emphasizing the importance of these residues in directing the decarboxylation pathway. Furthermore, the steady-state kinetic analysis of CYP-Aa162 and CYP-Sm46Δ29 revealed both cooperative and substrate inhibition behaviors which are substrate carbon chain length dependent.

**Conclusions:**

Our data identify CYP-Sm46Δ29 as an efficient OleT_JE_-like fatty acid decarboxylase. Oxidative decarboxylation chemoselectivity of the CYP152 decarboxylases is largely dependent upon the carbon chain length of fatty acid substrates and their precise positioning in the enzyme active site. Finally, the kinetic mode analysis of the enzymes could provide important guidance for future process design.

**Electronic supplementary material:**

The online version of this article (doi:10.1186/s13068-017-0894-x) contains supplementary material, which is available to authorized users.

## Background

Cytochrome P450 (CYP) enzymes are a superfamily of important biocatalysts that perform an extraordinary breadth of biochemical reactions [1–3], and have been widely applied to oxidation of complex organic substrates and biosynthesis of chemical building block molecules [4–6]. Among diverse P450 products, terminal alkenes (i.e., α-alkenes or 1-alkenes) have significant industrial potential and outstanding economic importance, given that these hydrocarbons highly mimic the chemical composition and physical properties of fossil fuels [7–10]. There has been widespread biotechnological interest focusing on the identification and reconstitution of enzymes and pathways capable of synthesizing biohydrocarbons in microbial hosts [7, 9, 11–17]. A sustainable biosynthetic route to 1-alkenes from biologically abundant feedstocks such as free fatty acids (FFAs) clearly represents a promising pathway. In 2011, Rude et al. [13] reported the first one-step enzymatic oxidative decarboxylation of FFAs by a P450 peroxygenase OleT_JE_, isolated from *Jeotgalicoccus* sp. ATCC 8456, to yield long-chain (C_13_–C_19_) 1-alkenes.

**Scheme 1:**
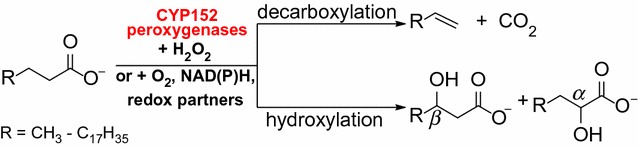
Fatty acid decarboxylation and hydroxylation catalyzed by CYP152 peroxygenases

In view of its tremendous potential for biological production of terminal olefins, OleT_JE_ since its discovery has captured great attention within academia and industry. A fast growing number of studies have been carried out to optimize OleT_JE_ catalysis and to understand its catalytic mechanism [16, 18–28]. These include the development of alternative catalytic systems (e.g., the O_2_/NAD(P)H/redox partners system [16] and the light-driven H_2_O_2_ generation system [21]) (Scheme 1), the determination of OleT_JE_ crystal structures [18, 28], the elucidation of catalytically reactive species [19, 20], and the expansion of substrate scope to structurally different aromatic carboxylic acids [29], dionic acids (to produce dienes) [30], and even some unnatural substrates including styrene, nonane, and cyclohexane [31].

Mechanistically, a majority of P450s use O_2_ and NAD(P)H-dependent redox systems to catalyze diverse oxidative/monooxygenation reactions [32, 33]. However, there are a small number of P450s that evolved to naturally utilize H_2_O_2_ as the sole oxygen and electron donor to catalyze oxidative reactions through the so-called peroxide shunt pathway [33–35]. These enzymes are referred to as P450 peroxygenases. The best-characterized P450 peroxygenases are among the CYP152 [36] family members, including P450_BSβ_ (CYP152A1) from *Bacillus subtilis* [35] and P450_SPα_ (CYP152B1) from *Sphingomonas paucimobilis* [34]. OleT_JE_, based on its amino acid sequence similarity, was designated as CYP152L1 [13, 18]. Within the CYP152 members that have been biochemically characterized so far, P450_SPα_ catalyzes exclusively the Cα hydroxylation of FFAs [34], and P450_BSβ_ generates both α- and β-hydroxy fatty acids as major products but with a small amount of 1-alkene as well [13, 35, 37]. A recently characterized CYP-MP peroxygenase [24] is able to introduce the hydroxyl group at α-, β-, γ-, δ-, and ε-positions of C_12_–C_18_ fatty acids, but it only displayed marginal decarboxylation activity against myristic acid (C_14_) and palmitic acid (C_16_). Heretofore, OleT_JE_ is the only one to predominantly catalyze the decarboxylation of long-chain fatty acids generating 1-alkenes as the major products and α- and β-hydroxy fatty acids as the side products (Scheme 1) [13, 16, 22]. It would be of great interest and importance to explore more P450 fatty acid decarboxylases and expand the enzyme inventory for future broader biotechnological applications.

In an attempt to screen for biocatalysts capable of converting medium-chain C_12_ fatty acid (lauric acid) to 1-undecene for potential applications in biofuels and biolubricants, two new P450 sequences, CYP-Aa162 (CYP152A8, GenBank Accession Number: WP_008340313) from *Alicyclobacillus acidocaldarius* LAA1 and CYP-Sm46 (CYP152L2, GenBank Accession Number: EKU50422) from *Staphylococcus massiliensis S46*, were identified apart from OleT_JE_ and P450_BSβ_. To better harness these two uncharacterized CYP152 family members for future industrial applications, and to further our understanding of the unique decarboxylation mechanism employed by CYP152 decarboxylases, we sought to characterize the detailed biochemical properties of the two enzymes.

In this study, we cloned, expressed, and purified CYP-Aa162 and CYP-Sm46 enzymes and in vitro characterized their catalytic activities towards a panel of different carbon chain length fatty acid substrates. Analytical data demonstrated that CYP-Aa162 acts mainly as a P450_BSβ_-like fatty acid hydroxylase, whereas CYP-Sm46 acts as an efficient OleT_JE_-like fatty acid decarboxylase. Their substrate conversion rates and regio- and chemoselectivity exhibited significant substrate carbon chain length dependence. In addition, our enzymatic kinetic analysis suggested that the metabolism of fatty acids by the CYP152 enzymes could undergo both cooperative and substrate inhibition behaviors, providing important information and guidance for future process design.

## Results

### Heterologous expression and purification of CYP-Aa162 and CYP-Sm46Δ29

According to protein sequence alignment (Additional file 1: Figure S1), CYP-Aa162 has 64% (39%) sequence identity to P450_BSβ_ (OleT_JE_), while CYP-Sm46 is 65% (37%) identical to OleT_JE_ (P450_BSβ_). Heterologous expression of CYP-Aa162 in *E. coli* (with its encoding sequence codon-optimized) led to an appreciable level of the recombinant protein purified to near homogeneity (Additional file 1: Figure S2). However, for CYP-Sm46, the original construct of pET28b-*CYP*-*Sm46* (codon-optimized) showed very poor expression and solubility, which did not improve upon subcloning into an alternative expression vector pCWori [38] or by optimization of the expression conditions. This prompted us to re-examine the protein sequence of EKU50422 and its annotation. We found that this sequence has an extra long (~29 amino acids) *N* terminus with no functional annotations when compared with most other CYP152 members including those cytochrome P450 family proteins from other *Staphylococcus* species (Additional file 1: Figure S1). Analyses of the CYP-Sm46 protein sequence using several protein prediction tools such as Phobius, TMHMM 2.0, and SignalP 4.1 [39–41] also showed no positive return of either a signal peptide or transmembrane helices within the *N*-terminal region of 60 amino acids. A further BLAST search revealed a recently added (17-Jan-2015) ‘truncated’ entry of CYP-Sm46 (GenBank Accession Number: WP_039990689), hereafter called CYP-Sm46Δ29, which decodes from an alternative start codon (GUG) and therefore has the 29 *N*-terminal amino acids removed. Subsequent expression of this shorter version CYP-Sm46Δ29 led to significantly improved protein production and solubility, and the protein was then purified to near homogeneity (Additional file 1: Figure S2). The codon-optimized gene sequences of CYP-Aa162 and CYP-Sm46Δ29, and their corresponding amino acid sequences are shown in Additional file 2.

### UV–visible spectroscopic properties of CYP-Aa162 and CYP-Sm46Δ29

**Fig. 1:**
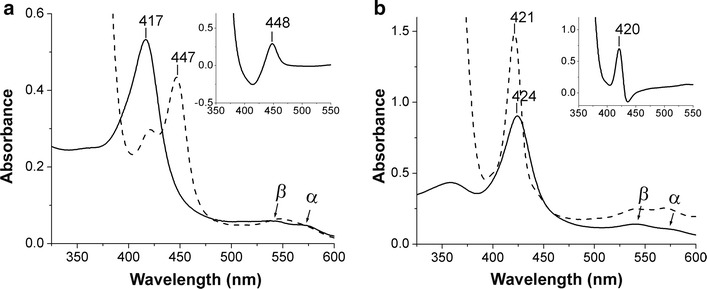
UV–visible spectra of CYP-Aa162 (**a**) and CYP-Sm46Δ29 (**b**). The purified enzymes were diluted in 50 mM NaH_2_PO_4_ (pH 7.4) containing 300 mM NaCl and 10% glycerol, respectively. Spectra are shown for the oxidized ferric form of the CYPs (*solid lines*) and the Na_2_S_2_O_4_-reduced ferrous-CO complex of the CYPs (*dashed lines*). *Insets* show the reduced CO difference spectrum of each enzyme

The resting ferric form of CYP-Aa162 (Fig. 1a) exhibited a typical low-spin (LS) water-bound P450 heme spectrum with its Soret absorption peak at 417 nm and the smaller α- and β-bands in the visible region at ~566 and ~538 nm, respectively. These values are similar to those of OleT_JE_ (418, 535, 566 nm) [18] and other LS bacterial P450s (e.g., the heme domain of CYP102A1 (P450_BM3_) from *Bacillus megaterium* with maxima at 418, 534, and 568 nm) [42]. However, CYP-Aa162 displayed no obvious hyperporphyrin (split Soret) characteristic at ~360 nm, which is different from that of OleT_JE_ [18]. A widely accepted criterion for the identification of cytochrome P450 is a Soret peak at around 450 nm in the reduced CO difference spectrum. Here, for CYP-Aa162, both the reduced CO difference spectrum and the absolute absorption spectrum of the dithionite-reduced Fe^2+^–CO adducts featured a characteristic thiolate-ligated P450 Soret band at 447–448 nm (Fig. 1a and inset), which is similar to that of P450_BSβ_ with a peak at 446 nm [35].

However, the purified ferric form of CYP-Sm46Δ29 displayed a red-shifted Soret band at 422–424 nm, followed by a small but prominent β band at 540 nm and a significantly weaker and flat α band at 571 nm (Fig. 1b). Prominent hyperporphyrin characteristics (split Soret) are visible at 360 nm. These features are similar to those reported for CYP-MP (maxima at 422, 545, and 575 nm) [24], but different from the typical 418, ~535, and ~568 nm maxima for LS water-bound P450s [18, 42]. These altered spectroscopic features may indicate an imidazole-coordinated or more likely a distal hydroxyl-coordinated ferric LS heme form as suggested in the CYP-MP [24]. Furthermore, much to our surprise, reduction of the CYP-Sm46Δ29 with sodium dithionite in the presence of CO revealed no characteristic P450 peak at ~450 nm. The maximum absorption of this Fe^2+^–CO adduct was still at ~421 nm with enhanced α- and β-bands. Correspondingly, the reduced CO difference spectrum of CYP-Sm46Δ29 also exhibited only the P420 peak (Fig. 1b inset). Attempts to produce a detectable P450 species by altering the amount of dithionite, buffer pH, and by substrate-aided stabilization of any, if exists, P450-CO adduct as the case with P450 EpoK enzyme [43] all proved negative (Additional file 1: Figure S3). The similar ‘P420’ spectra were observed in all preparations of the enzyme unanimously. These data suggest that CYP-Sm46Δ29 is somehow incapable of forming a detectable cysteine thiolate-ligated Fe^2+^–CO adduct. We speculate that this unusual spectroscopic feature may arise either from simple protonation of the proximal cysteine thiolate to a neutral thiol form [43, 44], or from dissociation of the thiolate ligand from the heme iron, followed by coordination of an alternative proximal ligand such as a histidine in the case with the pressure-induced P420_cam_ [45, 46], or from other reduction-linked structural alterations that prevent the formation of thiolate-ligated ferrous-CO species. Nevertheless, these altered spectroscopic features did not affect the enzyme’s peroxygenase activity as shown below.

### Inability of CYP-Aa162 and CYP-Sm46Δ29 to elicit monooxygenase activity

**Fig. 2:**
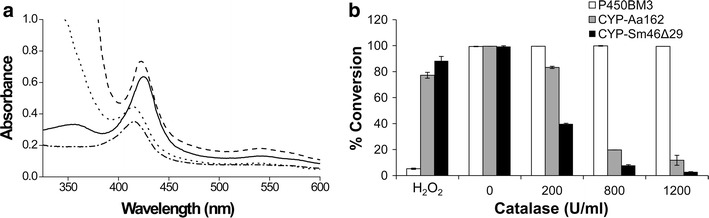
UV–visible spectra and lauric acid (LA) conversion activity of CYP-Aa162 and CYP-Sm46Δ29 in the O_2_/redox partners/NAD(P)H system. **a** The UV–visible spectra are, respectively, shown for the substrate LA-bound ferric form of CYP-Sm46Δ29 with a Soret band at 422 nm (*solid line*); the CamAB/NADH ‘reduced’ CO-bound form of CYP-Sm46Δ29 with the Soret peak still at ~422 nm (*dashed line*); the substrate LA-bound ferric form of CYP-Aa162 with a Soret band at ~418 nm (*dashed and dotted line*); the CamAB/NADH ‘reduced’ CO-bound form of CYP-Aa162 with an unshifted Soret peak (*dotted line*). **b** Enzymatic conversion of lauric acid by CYP-Aa162, CYP-Sm46Δ29, and the control P450_BM3_ monooxygenase in the O_2_/redox proteins/NAD(P)H system in the absence or presence of different concentrations of catalase. For CYP-Aa162 and CYP-Sm46Δ29, CamAB/NADH was used for the electron transfer cascade. For the self-sufficient P450_BM3_ enzyme, NADPH was used as the electron donor. Conversions of the substrate in the H_2_O_2_ cofactor system were included as controls

To check if a class I electron transfer system could afford a P450 Soret feature in CYP-Aa162 and CYP-Sm46Δ29 [47], we examined the UV–visible spectra of the two enzymes in an NAD(P)H/redox proteins/CO system. As a result, while the control P450 monooxygenase P450_BM3_ was successfully reduced by the addition of NADPH and exhibited an almost complete transition to P450 spectrum in the presence of lauric acid (LA) substrate (Additional file 1: Figure S4), neither CYP-Aa162 nor CYP-Sm46Δ29 showed a P450 chromophore after incubation with the redox partner proteins CamAB (i.e., putidaredoxin reductase and putidaredoxin that are able to support the in vitro activity of both P450 enzymes, see below) and their optimal cofactor NADH in the presence of LA (Fig. 2a). Further catalytic activity assay indicated that the two enzymes exhibited appreciable LA conversion activity in the H_2_O_2_ system. However, their activity was dramatically diminished in the O_2_/CamAB/NADH reaction system when high concentrations of catalase were added to remove H_2_O_2_ potentially generated from the decoupling of NADH oxidation (Fig. 2b), implying that CYP-Aa162 and CYP-Sm46Δ29 have very little or no monooxygenase activity. The marginal residual activity of the two enzymes shown at 1200 U mL^−1^ catalase condition may represent some inevitable leakage of H_2_O_2_ to the P450 proteins, as even in the absence of putidaredoxin there was still meager activity detectable at this high catalase concentration (Additional file 1: Figure S5).

### Substrate specificity and chemoselectivity of CYP-Aa162 and CYP-Sm46Δ29

Members in the CYP152 peroxygenase family are capable of utilizing H_2_O_2_ as the sole electron and oxygen donor to carry out catalytic reactions [33]. Among those CYP152s that have been biochemically characterized so far, OleT_JE_ catalyzes predominantly the decarboxylation of long-chain fatty acids to give 1-alkenes as the main products and α- and β-hydroxy fatty acids as the side products [13]. Other CYP152 enzymes including P450_SPα_, P450_BSβ_, and CYP-MP catalyze primarily the hydroxylation of free fatty acids, introducing the hydroxyl group at varying carbon positions (i.e., α-, β-, γ-, δ-, or ε-position) [24, 34, 35, 37]. Here, in order to investigate the substrate specificity and chemoselectivity (decarboxylation vs hydroxylation) of the two new CYP152 enzymes, we set out to examine their catalytic reactions towards a range of different carbon chain length (C_10_–C_20_) FFAs using H_2_O_2_ as the sole cofactor.

**Fig. 3:**
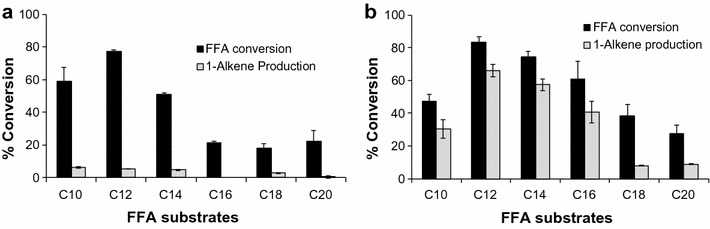
Substrate preference and 1-alkene production profiles of CYP-Aa162 (**a**) and CYP-Sm46Δ29 (**b**). Substrate preference was determined by calculating the percentage conversion of each fatty acid substrate. The corresponding 1-alkene production represents the percentage of 1-alkene yield over the starting substrate. Results shown are mean ± SD of duplicated experiments

As a result, both enzymes exhibited quite similar substrate preference profiles with lauric acid (C_12_) being the best substrate (Fig. 3). Its conversion ratios were 77.3 ± 1.0 and 83.5 ± 3.4% by CYP-Aa162 and CYP-Sm46Δ29, respectively. In addition, CYP-Sm46Δ29 also converted myristic acid (C_14_) efficiently (74.5 ± 3.5%). However, in terms of decarboxylation, CYP-Aa162 showed only marginal alkene production from its best substrate C_12_ (5.1 ± 0.1%), whereas CYP-Sm46Δ29 exhibited significantly higher percentage of alkene production. The optimal substrates C_12_ and C_14_ FFAs reached the alkene production efficiency of 66.1 ± 3.9 and 57.4 ± 3.6%, respectively. Unlike previously reported for OleT_JE_ by others [13, 21], CYP-Sm46Δ29 favors more the medium-chain length fatty acids (C_12_ or C_14_) rather than the long ones (C_16_–C_20_). The decarboxylation activity of CYP-Sm46Δ29 (Fig. 3) and the ratio of decarboxylation over hydroxylation activities (Additional file 1: Figure S6) gradually reduced after C_12_ with the increase in substrate carbon chain length, and the hydroxyl FAs became the major products in the reactions with C_18_ or C_20_ as a substrate, indicating that different substrate binding modes are likely employed by this enzyme.

**Fig. 4:**
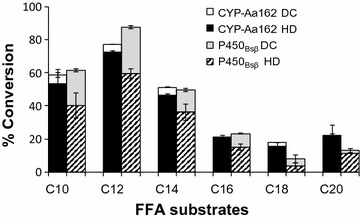
Decarboxylation (DC) versus hydroxylation (HD) activities of CYP-Aa162 and P450_BSβ_. The hydroxylation activity was estimated by subtracting the alkene production from the total substrate conversion. This indirect but more convenient method was validated with C_14_ myristic acid substrate by direct measurement of its derivatized hydroxylation products. Results are shown as mean ± SD of duplicated experiments

In consideration of the high sequence identity (64%) and similar UV–visible absorption maxima between CYP-Aa162 and P450_BSβ_, we compared their catalytic activities in parallel. As shown in Fig. 4, the two enzymes exhibited very similar substrate preference spectra and total fatty acid conversion efficiency. However, P450_BSβ_ showed generally higher alkene production ratios compared with CYP-Aa162. The best alkene-producing substrate lauric acid (C_12_) displayed a fivefold higher 1-undecene production (27.7 ± 0.9% versus 5.1 ± 0.1%). Nonetheless, substrate hydroxylation still represented the major chemistry of the two enzymes, which is not surprising since P450_BSβ_ was initially identified as a P450 fatty acid hydroxylase [35]. The hydroxylation versus decarboxylation ratios of the two CYPs towards their preferred C_10_–C_14_ substrates were at least 2.5:1. Thus, we designated CYP-Aa162 as a P450_BSβ_-like fatty acid hydroxylase rather than a P450 decarboxylase.

Furthermore, to comparatively investigate the detailed product distribution, the C_14_ substrate myristic acid was reacted with the four enzymes CYP-Aa162, CYP-Sm46Δ29, P450_BSβ_, and OleT_JE_, respectively. The results (Table 1) revealed that CYP-Aa162 formed predominantly (78.9 ± 1.4%) the α-hydroxy myristic acid (α-OH–C_14_), and P450_BSβ_ exhibited approximately even distribution among the three products α-OH–C_14_, β-OH–C_14_, and 1-tridecene. In comparison, CYP-Sm46Δ29 and OleT_JE_ showed strong decarboxylation chemoselectivity. For these two enzymes, 1-tridecene was the major product, β-OH–C_14_ the minor product, while α-OH–C_14_ only accounted for a negligible proportion of the total products. In all cases, no distal hydroxylated fatty acids beyond C_β_ position were detected, as opposed to the mixed regioselectivity found with the CYP-MP enzyme [24]. Together, these data strongly support the notion that CYP-Aa162 functions as a P450_BSβ_-like fatty acid hydroxylase, while CYP-Sm46Δ29 acts as an efficient OleT_JE_-like decarboxylase, providing another enzyme to the unusual fatty acid oxidative decarboxylation reaction (Additional file 1: Figure S7).

**Table 1:** GC–MS analysis of catalytic activities and product distribution profiles of the selected CYP152 peroxygenases towards myristic acid (C_14_)

Enzymes	Conversion (%)	Product distribution (%)		
		1-tridecene	α-OH–C_14_	β-OH–C_14_
CYP-Aa162	60.1 ± 2.1	16.0 ± 1.2	78.9 ± 1.4	5.1 ± 0.6
P450_BSβ_	62.8 ± 5.0	36.8 ± 3.5	30.0 ± 4.2	33.2 ± 3.5
CYP-Sm46Δ29	73.2 ± 8.9	78.4 ± 2.9	0.4 ± 0.1	21.2 ± 0.9
OleT_JE_	74.2 ± 2.9	91.8 ± 5.9	0.3 ± 0.1	7.9 ± 1.5

### Site-directed mutagenesis of CYP-Aa162 and P450_BSβ_ fatty acid hydroxylases

The active-site sequence alignment of CYP-Aa162 and CYP-Sm46Δ29 with those of P450_BSβ_ and OleT_JE_ revealed two major residue differences (Additional file 1: Figure S8): the histidine and isoleucine residues in the FFA decarboxylases of OleT_JE_ (H85 and I170) and CYP-Sm46Δ29 (H86 and I171) are replaced by a glutamine and a valine in CYP-Aa162 (Q85 and V170) and P450_BSβ_ (Q85 and V170), respectively. According to the crystal structure of OleT_JE_ (PDB: 4L40) [18], H85 and I170 are positioned at the two sides of the substrate carboxyl group with the distance of 5.1 and 3.4 Å, respectively. Previously, Rude et al. [13] reported an improved decarboxylation activity (~50%) of P450_BSβ_ Q85H variant towards palmitic acid (C_16_), suggesting an important role of this histidine residue in regulating the decarboxylation chemoselectivity of OleT_JE_. Grant et al. [20] also proposed mechanisms in which H85 of OleT_JE_ may act as a proton donor to facilitate the protonation of OleT_JE_-Compound II intermediate (Fe^4+^–OH) generated from substrate C–H abstraction and thereby to initiate substrate C–C_α_ bond scission (i.e., decarboxylation). Moreover, our recent saturation mutagenesis analysis of OleT_JE_ at H85 and I170 sites also confirmed the importance of these two amino acid residues for decarboxylation activity and substrate positioning based on the finding that all H85X (X denotes any natural amino acid) and I170X mutants exhibited abolished decarboxylation activity towards myristic acid [26].

**Fig. 5:**
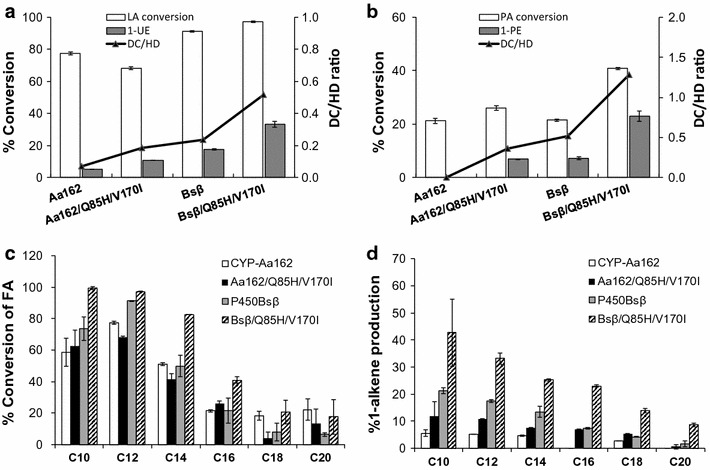
Fatty acid conversion by the Aa162/Q85H/V170I and P450_Bsβ_/Q85H/V170I mutants compared to their corresponding wild-type enzymes. The hydroxylation (HD) activity was deduced by subtracting the 1-alkene production (corresponding to DC activity) from the total substrate conversion. **a** Lauric acid (LA) conversion and 1-undecene (1-UE) production; **b** palmitic acid (PA) conversion and 1-pentadecene (1-PE) production; **c** conversion ratios of different carbon chain length fatty acids (C_10_–C_20_) and **d** their corresponding 1-alkene production by the indicated enzymes. Results are presented as mean ± SD of duplicated experiments

Therefore, in view of the potential key roles of these two residues in regulating decarboxylation process and substrate positioning, we decided to substitute the corresponding amino acids in CYP-Aa162 and P450_Bsβ_ with His and Ile, and created the double mutants Aa162/Q85H/V170I and P450_Bsβ_/Q85H/V170I. Their in vitro activities towards a series of free fatty acid substrates (C_10_–C_20_) were evaluated along with their corresponding wild types. As a result, the P450_Bsβ_/Q85H/V170I mutant exhibited a general increase in the total substrate conversion ratios compared with its wild type, while Aa162/Q85H/V170I showed variations of increase or decrease in conversion efficiency towards different carbon chain length substrates (Fig. 5c). However, both mutants displayed enhanced decarboxylation activity to the tested substrates to different extents (Fig. 5d). This increase was more prominent in the longer carbon chain length substrates such as C_16_ palmitic acid (Fig. 5b) than in the shorter carbon chain length substrates such as C_12_ lauric acid (Fig. 5a). For C_12_ substrate, a 1.1- and a 0.9-fold increase in 1-undecene production were observed with Aa162/Q85H/V170I and P450_Bsβ_/Q85H/V170I mutant, accounting for a 1.7- and a 1.2-fold increase in the ratio of decarboxylation over hydroxylation activity, respectively (Fig. 5a). For C_16_ substrate, the production of 1-pentadecene exhibited an increase from undetectable by the wild type CYP-Aa162 to 6.9% by its double mutant Aa162/Q85H/V170I, and an increase of 2.1-fold by the P450_Bsβ_/Q85H/V170I mutant when compared with its wild type P450_Bsβ_. The latter increase led to a high decarboxylation versus hydroxylation ratio of around 1.3 in the P450_Bsβ_/Q85H/V170I mutant, making the 1-alkene the major product. Together, these results demonstrate the important role of the active-site histidine and isoleucine in regulating fatty acid decarboxylation activity of the CYP152 peroxygenases.

### Steady-state kinetics of CYP-Aa162 and CYP-Sm46Δ29

To better understand the catalytic properties of CYP-Aa162 fatty acid hydroxylase and CYP-Sm46Δ29 fatty acid decarboxylase, we determined their steady-state kinetic parameters (Table 2) towards their optimal substrates C_12_ and/or C_14_ fatty acids. Since CYP-Aa162 mainly catalyzes the hydroxylation of fatty acids, trace amounts of alkene products were undetectable at the beginning of the reaction when initial rates were determined. Therefore, its kinetic constants were derived from the rates of substrate consumption. For CYP-Sm46Δ29, the kinetic constants were specifically determined by 1-alkene formation rates due to its much higher decarboxylation activity. Interestingly, it was revealed that the kinetic data of both CYP-Aa162 and CYP-Sm46Δ29 towards C_12_ substrate fit well to a sigmoidal Hill equation with a Hill coefficient value around 2 (Table 2; Additional file 1: Figure S9A, B). This may suggest that the substrate binding pocket of the two P450 enzymes could accommodate more than one C_12_ molecules and that the binding of one C_12_ molecule could facilitate the binding of the other to the same P450 enzyme, particularly at low to medium substrate concentrations (10–80 μM). Notably, the kinetic data of CYP-Sm46Δ29 towards C_14_ substrate exhibited apparent and substantial substrate inhibition (Additional file 1: Figure S9C). When the substrate concentration was in excess of 60 μM, the rate of 1-tridecene formation declined dramatically, but was not completely abolished even at a high concentration of 300 μM. Rather, it flattened off to a low activity, indicating an incomplete inhibition. Estimation of the kinetic parameters for this substrate was therefore deduced from the truncated dataset using Michaelis–Menten equation after removal of the inhibited rates at high substrate concentrations [48]. The apparent *k*_*cat*_ value of 62.5 ± 4.5 min^−1^ is comparable to that of OleT_JE_ towards C_14_ substrate (71.0 ± 8.4 min^−1^) [26]. Collectively, these data imply that, to different carbon chain length FFA substrates, the decarboxylase CYP-Sm46Δ29 could present distinct binding modes and therefore display different kinetic behaviors.

**Table 2:** Kinetic parameters of FFA conversion catalyzed by CYP-Aa162 and CYP-Sm46Δ29

Enzymes	C_12_^a^				C_14_^b^		
	*k*_*cat*_ (min^−1^)	*K*_*H*_ (uM)	*K*_*cat*_/*K*_*H*_ (uM^−1^ min^−1^)	Hill coefficient (n)	*k*_*cat*_ (min^−1^)	*K*_*m*_ (uM)	*K*_*cat*_/*K*_*m*_ (uM^−1^ min^−1^)
CYP-Aa162^c^	46.1 ± 17.1	93.1 ± 37.7	0.5	2.1 ± 0.8	nd	nd	nd
CYP-Sm46Δ29^d^	24.5 ± 6.3	58.4 ± 19.0	0.4	2.2 ± 1.0	62.5 ± 4.5	41.3 ± 5.1	1.5

*nd* not determined

^a^Data were fitted to Hill equation ($$v = {\raise0.7ex\hbox{${V_{ \hbox{max} } \left[ {\text{S}} \right]^{n} }$} \!\mathord{\left/ {\vphantom {{V_{ \hbox{max} } \left[ {\text{S}} \right]^{n} } {\left( {K_{H}^{n} + \left[ {\text{S}} \right]^{n} } \right)}}}\right.\kern-0pt} \!\lower0.7ex\hbox{${\left( {K_{H}^{n} + \left[ {\text{S}} \right]^{n} } \right)}$}},$$ *n* Hill coefficient)

^b^Substantial substrate inhibition was observed with this substrate. Data shown here were derived from Michaelis–Menten equation after omitting the inhibited rates at high substrate concentrations

^c^Initial rates were measured by the amount of substrate consumption per μM enzyme per min

^d^Initial rates were measured by the amount of 1-alkene formation per μM enzyme per min. Data (shown as mean ± SE) were calculated using OriginPro 8.0 program

## Discussion

In this study, we successfully expressed, purified, and biochemically characterized two new CYP152 peroxygenase family members, CYP-Aa162 and CYP-Sm46Δ29, that are capable of decarboxylating straight-chain saturated fatty acids to yield 1-alkenes to different extents. Previously, Chen et al. [49] compared the in vivo alkene production by expressing a His-tagged CYP-Sm46 (there called OleT_SM_, GenBank Accession Number: WP_009381667, an identical sequence to EKU50422, 453 amino acids) in *S. cerevisiae*, and only observed trace amounts of odd-numbered carbon chain alkenes (C_13_, C_15_, and C_17_) from a 48-h culture of the recombinant yeast cells (total alkene titer 1.4 μg L^−1^, 40 times lower than that of OleT_JE_ 54.5 μg L^−1^). In that study, a ‘full-length’ version of CYP-Sm46 was used. No protein expression data were shown, nor was enzymatic characterization performed. Here, our experiment in *E. coli* proved very poor or no soluble expression of the ‘full-length’ version of CYP-Sm46. Translation in bacteria is most commonly initiated at an AUG start codon, which encodes for a methionine. However, in some species, the start codon can be GUG, which within the open reading frame (ORF) would encode for a valine. *Staphylococcus aureus* has been documented to use GUG as an alternative start codon [50]. The use of alternative initiation codons can influence translation efficiency, while incorrect initiation could lead to misfolded and therefore inactive proteins. The initial amino acid sequence of CYP-Sm46 (EKU50422) was most likely mistakenly derived from the usual AUG start codon as it happens to follow a putative Shine–Dalgarno sequence (AGGATG), which may explain why we did not observe decent expression with the original EKU50422 sequence. The later version of the amino acid sequence (CYP-Sm46Δ29) adopted GUG as its start codon (WP_039990689), and a possible Shine–Dalgarno site of 5′-TGGGGG-3′ was found 7 base upstream of the putative GUG start codon in the mRNA sequence (Additional file 2). The RBS calculator 2.0 (http://www.denovodna.com/software/forward) analysis of the CYP-Sm46 mRNA sequence showed that the GUG has a fivefold higher translation rate (3.3-fold lower total initiation energy ΔG_Total_) than that of the upstream AUG start codon. In addition, McLaughlin et al. [51] reported the identification of a very strong Shine–Dalgarno complementarity containing five G-C base pairs in the *Staphylococcus aureus* β-lactamase gene. This strong mRNA-16S rRNA binding was proposed to reduce dependence on other initiation factors in the Gram-positive bacteria such as *Bacillus* and *Staphylococcus*. Furthermore, analysis of mRNA  sequences of cytochrome P450 family proteins from other *Staphylococcus* species such as *S. agnetis* (GenBank: JPRT01000001.1) and *S. pseudintermedius* (GenBank: CP002439.1) also revealed a similar pattern of the strong 5–6 G-C pairs of Shine–Dalgarno complementarity preceding a GUG start codon. Taken together, we reason that CYP-Sm46Δ29 is most likely to be the native functional form of the protein.

P420 species was generally thought to be an inactive form of cytochrome P450 enzymes [52–54]. Formation of this ‘inactive’ P420 was suggested to involve the protonation of the native cysteine thiolate to a neutral thiol heme ligand [44], the recruitment of a histidine to replace the native cysteinate ligand [45], or other unknown reasons. Either way leads to the failure of forming the thiolate-ligated Fe^2+^ species or to a distorted/weakened heme–thiolate bond, thus underlying the P450 → P420 transition [45]. Most P420 studies were performed with the P450_BM3_ [52, 54] or P450_cam_ [45, 53] monooxygenases. These enzymes employ redox partner system to transfer electrons from NAD(P)H and activate O_2_ as the oxidant, whose catalytic activity depends on the formation of a specific thiolate bond between the heme iron and the absolutely conserved cysteine residue in order to afford an active conformation state [33]. P450 peroxygenases, on the other hand, unlike those monooxygenases, utilize peroxide (such as H_2_O_2_) to directly convert the heme iron of enzymes to a reactive ferric-hydroperoxy complex (Fe^3+^–OOH) via the peroxide shunt pathway [33, 36], therefore bypassing the oxygen activation chemistry and the necessity of forming the thiolate-ligated ferrous-heme species. Whether the failure of observing a P450 species in CYP-Sm46Δ29 is due to the instability of the thiolate-ligated P450 species that quickly reverts to a P420 form as with EpoK [43], or an alternative proximal ligand-coordinated species as in P420_cam_ [45], P450 enzymes that undergo peroxide shunt pathway are insensitive to the nature of the proximal ligand as they do not require electron transfer to the heme iron. Peroxide activation can be achieved equally well with, for example, the histidine-ligated hemoproteins [55]. Therefore, as opposed to the generally regarded ‘inactive’ cytochrome P420 species in the P450 monooxygenases, the peroxygenase ‘P420’ enzymes can be active. We reason that this difference may explain why we observed no thiolate-ligated P450 ferrous-heme species but a functional CYP-Sm46Δ29. Indeed, we observed efficient catalytic activity of CYP-Sm46Δ29 in the H_2_O_2_ system, but not in the H_2_O_2_-depleted O_2_/redox proteins/NADH system (Fig. 2b). A minor increase in the activities of CYP-Aa162 and CYP-Sm46Δ29 was observed in the catalase-absent redox partner-supported reaction system (Fig. 2b). This increase may reflect the beneficial effect of slow-releasing H_2_O_2_ from NADH decoupling on the catalytic activity, compared with the batch-added H_2_O_2_ [24]. However, considering the unique spectroscopic features of the CYP-Sm46Δ29, further structural and mechanistic studies are certainly required to gain more detailed understanding of the active-site coordination environment of this enzyme and its catalytic mechanism.

The strict carbon chain length dependence of the decarboxylative activity of CYP-Sm46∆29 is consistent with other identified decarboxylative activities in OleT_JE_ [13, 16, 22], P450_BSβ_ [37], and CYP-MP [24], which further emphasizes the importance of substrate identity in controlling the hydroxylation/decarboxylation bifurcation. This substrate identity-dependent chemistry was also manifested in a recent study of OleT_JE_ where only trace amounts (<1–2%) of undesired hydroxyl side products were generated when aromatic carboxylic acids were used as substrates [29]. Our finding that low alkene production in CYP-Aa162 is accompanied with a high percentage of α-hydroxyl product is also consistent with the notion that C_β_–H rather than C_α_–H abstraction is a prerequisite for initiation of fatty acid decarboxylation pathway. However, this could not be the sole determining factor as CYP-MP produced predominantly the β-OH fatty acid but only a meager amount of alkenes [24]. For OleT_JE_, it was proposed that His85 could facilitate the decarboxylation pathway through providing proton to the Fe^4+^–OH (compound II) intermediate to restore a water-bound Fe^3+^–OH_2_ species [18, 20]. An active-site histidine may also help with the precise substrate positioning required for the decarboxylation pathway [26]. In the absence of an active-site histidine, ∙OH rebound would be the prevalent pathway, leading to accumulated hydroxyl fatty acids, such as the case with CYP-MP [24], P450_Bsβ_ [13], and CYP-Aa162 in this study. Our site-directed mutagenesis analysis of CYP-Aa162 and P450_Bsβ_ confirmed the importance of this residue in regulating decarboxylation selectivity. However, the introduction of this histidine residue did not fully reverse the chemoselectivity of either CYP-MP [24] or CYP-Aa162 (Fig. 5), albeit an increase in the decarboxylation activity, hydroxylation was still the major chemistry of CYP-Aa162/Q85H/V170I. This implies that more structural and electronic requirements need to be satisfied to be a fatty acid decarboxylase.

Makris and coworkers [19, 20] recently confirmed that OleT_JE_ catalysis is initiated by the formation of an iron(IV)-oxo cation radical (compound I). The fatty acid decarboxylation bifurcation is believed to begin with a regio-specific C_β_–H abstraction followed by a single-electron transfer from either a substrate carboxylate group or an additional electron from the C_β_ position to the incipient compound II, resulting in the generation of a substrate diradical or a substrate carbocation, respectively, to fulfill C–C_α_ bond scission and give rise to the terminal alkene products. Our data here together with others [13, 18–20, 23, 24] suggest that substrate C_β_–H abstraction, chain length-dependent precise positioning at the active site, relative intrinsic stability of the enzyme/Fe^4+^–OH intermediate (compound II), and the competition between ·OH rebound and abstraction of an additional substrate electron by compound II are among the decarboxylation bifurcation determinants to ensure a desaturation reaction. Given that site-directed mutagenesis did not fully boost the decarboxylation activity of CYP-Aa162, alternative approaches such as directed evolution may represent an attractive method for engineering improved alkene formation activity towards, especially, particular chain length substrates, and for thorough understanding of the enzyme structure–function relationship.

A majority of P450 catalyses follow simple Michaelis–Menten kinetics based on a single active site for the substrate–enzyme interaction [56]. However, there are growing cases that non-Michaelis–Menten kinetics were observed for some P450 reactions [48, 57–59]. In the latter cases, the non-classical kinetics apparently results from allosteric effects that commonly lead to a sigmoidal velocity curve. The Hill equation can be used to indicate cooperative kinetics. Indeed, several studies have found that some P450s are able to fit into its active site with large molecules such as cyclosporine [60] or two substrates at once [57]. The two substrates can be the same or different molecules allowing the homotropic or heterotropic cooperative kinetics, respectively. Other examples of cooperative catalysis were found with P450_SPα_ [61, 62] and P450_BSβ_ [62, 63] where short-chain length fatty acids (such as heptanoic acid or hexanoic acid) could act as ‘decoy molecules’ to facilitate the binding of non-natural substrates (such as styrene) and their oxidation by these P450 peroxygenases. In this study, cooperative binding of C_12_ lauric acid substrate to CYP-Aa162 and CYP-Sm46Δ29 enzymes was observed with a Hill coefficient of ~2 (Table 1). The smaller *k*_*cat*_ and *k*_*cat*_/*K*_*m*_ values of CYP-Sm46Δ29 towards C_12_ lauric acid (24.5 ± 6.3 min^−1^ and 0.4 μM^−1^ min^−1^) than those towards C_14_ myristic acid (62.5 ± 4.5 min^−1^ and 1.5 μM^−1^ min^−1^) may appear to be contradictory to the qualitative substrate specificity results (Fig. 2) which demonstrated higher conversion efficiency of C_12_ over C_14_ by this enzyme. We reason that this inconsistency may stem from the substrate inhibition of this enzyme by the high substrate concentration of C_14_ (200 μM substrate was used for the substrate specificity profiling assay).

## Conclusions

In this study, we have characterized two new CYP152 peroxygenases with regard to their spectroscopic characteristics, substrate specificity, decarboxylation/hydroxylation chemoselectivity, and kinetics. Our data demonstrate that CYP-Sm46Δ29 works as an efficient OleT_JE_-like fatty acid decarboxylase, while CYP-Aa162 acts as a P450_BSβ_-like fatty acid hydroxylase with marginal decarboxylation chemoselectivity. Furthermore, CYP-Sm46Δ29 was found to be catalytically active without forming a stable P450 species. Its decarboxylation activity exhibited strict carbon chain length dependence, suggesting the requirement of precise active-site positioning of substrates to be decarboxylated. Moreover, towards different carbon chain length fatty acids, CYP-Sm46Δ29 undertook different kinetic behaviors (cooperative or substrate inhibition), further stressing the importance and impact of elaborate coordination of substrate within the substrate binding pocket. Taken together, our findings could provide more opportunities for further understanding the unique catalytic mechanism employed by CYP152 fatty acid decarboxylases and also serve as a guide for future bioengineering of the enzymes for improved alkene-producing activity of different carbon chain length substrates.

## Methods

### Materials

Fatty acid substrates, terminal alkene authentic standards, α-hydroxy myristic acid, β-hydroxy myristic acid, and *N*,*O*-bis(trimethylsilyl)trifluoroacetamide (BSTFA) with 1% trimethylchlorosilane were purchased from TCI (Shanghai, China). Catalase from bovine liver (# C1345) was purchased from Sigma-Aldrich (St. Louis, MO, USA). One unit is defined, as described in the manufacturer’s instruction, to decompose 1.0 μmol of H_2_O_2_ min^−1^ at pH 7.0 at 25 °C, while the H_2_O_2_ concentration falls from 10.3 to 9.2 mM, measured by the rate of decrease of A_240_. NADH and antibiotics were obtained from Solarbio (Beijing, China). Other chemicals were purchased from Ameresco (Solon, OH, USA) or Sigma-Aldrich (St. Louis, MO, USA). Molecular cloning kits, such as High Purity Plasmid Miniprep Kit and Wizard SV Gel and PCR Clean-up System, were purchased from TSINGKE Biotech (Beijing, China) and Promega (Madison, WI, USA), respectively. Oligonucleotides and codon-optimized genes were synthesized by Genewiz (Suzhou, China). The *Pfu* DNA polymerases and all restriction endonucleases were obtained from Takara (Dalian, China). Ni–NTA resin used for protein purification was purchased from Qiagen (Valencia, CA, USA). The FlexiRun^TM^ pre-mixed gel solution for SDS-PAGE was obtained from MDBio, Amicon Ultra centrifugal filters from Millipore (Billerica, MA, USA), and PD-10 desalting columns from GE Healthcare (Piscataway, NJ, USA).

### Molecular cloning

The gene sequences encoding CYP-Aa162 from *Alicyclobacillus acidocaldarius* LAA1 (CYP152A8, GenBank Accession Number: WP_008340313) and CYP-Sm46 from *Staphylococcus massiliensis* strain *S46* (CYP152L2, GenBank Accession Number: EKU50422) were, respectively, codon-optimized and synthesized by Genewiz (Suzhou, China), and cloned into the expression vector pET28b between NdeI/XhoI restriction sites with a hexahistidine tag at the *N* terminus for expression in *E. coli* BL21 (DE3). The truncated *CYP*-*Sm46Δ29* gene (WP_039990689) was PCR-amplified from the initial construct of pET28b-*CYP*-*Sm46* using *Pfu* DNA polymerase and then subcloned into pET28b vector using the same restriction sites as above. The generation of double mutant gene constructs of Aa162/Q85H/V170I and P450_BSβ_/Q85H/V170I was achieved by site-directed mutagenesis via overlap extension PCR [64]. The sequences of primers used in this study are listed in Additional file 3: Table S1. All plasmid constructs were confirmed by DNA sequencing at Sangon Biotech (Shanghai, China). Upon sequence verification, plasmids were used to transform *E. coli* BL21 (DE3) for protein expression.

### Heterologous expression and purification

The *E. coli* BL21 (DE3) cells transformed with pET28b-*CYP*-*Sm46Δ29*, pET28b-*CYP*-*Aa162*, or the Q85H/V170I double mutant constructs were grown overnight at 37 °C with shaking at 220 rpm in LB medium containing 50 μg mL^−1^ kanamycin. The overnight culture was used as a seed culture to inoculate (1:100 dilution) 1–3 L of modified terrific broth containing 4% glycerol, 1 mM thiamine, trace metals [16], and the corresponding antibiotics. Cells were then grown at 37 °C for 3–4 h until the optical density at 600 nm (OD_600_) reached ~0.6, at which point *δ*-aminolevulinic acid (0.5 mM final concentration) was supplemented and the expression of CYP-Sm46Δ29 was induced by the addition of 0.2 mM isopropyl-β-d-thiogalactopyranoside (IPTG). Cells were further cultured for 24 h at 18 °C before being harvested by centrifugation at 6000 rpm, 4 °C. The cell pellet was frozen at −80 °C until required.

Purification of the His-tagged protein was carried out as described by Liu et al. [16] with minor modifications. All protein purification steps were performed at 4 °C. Specifically, the cell pellets were thawed and resuspended in 40 mL lysis buffer (50 mM NaH_2_PO_4_, 300 mM NaCl, 10% glycerol, and 10 mM imidazole, pH 8.0) through vortexing. After cell disruption by ultrasonication, the cell lysate was centrifuged at 12,000×*g* for 30 min to remove cellular debris. To the clarified cell lysate, 1 mL of Ni–NTA resin was added and gently mixed at 4 °C for 1 h. The slurry was then loaded onto an empty column and washed with approximately 100 mL of wash buffer (50 mM NaH_2_PO_4_, 300 mM NaCl, 10% glycerol, and 20 mM imidazole, pH 8.0) until no proteins were detectable in flowthrough. The bound target proteins were eluted with elution buffer (50 mM NaH_2_PO_4_, 300 mM NaCl, 10% glycerol, and 250 mM imidazole, pH 8.0). The eluates were pooled and concentrated with an Amicon Ultra centrifugal filter (30 kDa cutoff). Imidazole contained in the protein eluates was removed by ultrafiltration and buffer exchange on a PD-10 column into storage buffer (50 mM NaH_2_PO_4_, 300 mM NaCl, 10% glycerol, pH 7.4). The final purified protein was flash-frozen with liquid nitrogen and stored at −80 °C for later use.

### UV–visible spectroscopic characterization of CYPs

Analysis of the UV–visible spectroscopic properties of the His-tagged CYP-Aa162 and CYP-Sm46Δ29 was performed on a Cary 60 UV–visible spectrophotometer (Varian, UK). For preparation of the dithionite-reduced ferrous-CO complex of each enzyme, carbon monoxide gas was slowly bubbled into a solution of purified ferric enzyme (~4–7 μM) in 50 mM NaH_2_PO_4_, 300 mM NaCl, 10% glycerol, pH 7.4, immediately followed by sufficient reduction of the protein with sodium dithionite (1–3 mg) [65]. The optical absorption spectra of the ferric and ferrous-CO forms of each enzyme were recorded, respectively, before and after the addition of sodium dithionite. The CO-bound reduced difference spectrum was obtained according to the previous report [66]. In order to record the spectra in the redox partners/NAD(P)H/CO system, lauric acid (~0.7 mM) was pre-incubated with the cytochrome proteins for 5 min at 28 °C before the absorbance of ferric forms was recorded. Then, to the substrate-bound protein solution was added the CamAB/NADH (for CYP-Aa162 and CYP-Sm46Δ29) or NADPH (for the self-sufficient P450_BM3_) cofactors to initiate a flavin-to-heme electron transfer. Spectra of the CO-bound NAD(P)H-reduced forms of the P450 enzymes were subsequently recorded. The protein concentration was determined by the extinction coefficient of ε_422 nm_ = 104 mM^−1^ cm^−1^ as determined using the pyridine hemochromogen method [67]. The typical methodology for determination of the P450 functional concentration using ε_450–490 nm_ [66] was abandoned due to lack of the P450 content in the CYP-Sm46Δ29 spectra.

### In vitro enzymatic assay

Typical assays containing 1.5 μM of each CYP enzyme (CYP-Sm46Δ29 or CYP-Aa162 or their mutant), 200 μM fatty acid substrate (one of the C_10_–C_20_ FFAs prepared from a 20 mM stock solution in DMSO), and 220 μM H_2_O_2_ in 200 μL of storage buffer were carried out at 28 °C for 2 h. Reactions were quenched by the addition of 20 μL of 10 M HCl. Heptadecanoic acid was then added as the internal standard, and the mixture was extracted by 200 μL ethyl acetate. Following extraction, the organic phase was collected and analyzed by gas chromatography (GC) as described below.

For detection of the volatile C_9_ nonene product generated from C_10_ fatty acid decarboxylation, 500 μL of the reaction system containing 200 μM C_10_ fatty acid substrate, 220 μM H_2_O_2_, 2.0 μM of enzyme, and 200 μM C_7_ 1-heptene as the internal standard in a 1.5 mL polytetrafluorethylene (PTFE) septum-sealed glass bottles was incubated at 28 °C for 2 h with shaking at 300 rpm. The reactions were then placed on ice and subjected to headspace sampling using a gas-tight Hamilton syringe for GC–MS analysis as described below. The standard curve of nonene was obtained by incubating different concentrations of the authentic nonene standard under the same condition as the reactions without enzyme followed by the same GC–MS analysis. After headspace sampling, the reactions were immediately mixed with ethyl acetate and the internal standard heptadecanoic acid and extracted as described above for the analysis of the remaining C_10_ substrates. With every tested substrate (C_10_–C_20_ FFAs), a reaction without H_2_O_2_ was used as the control for the initial substrate concentration.

### Steady-state kinetic analysis

To determine the P450 kinetic parameters, 0.1–0.5 nM of enzyme was incubated with varying concentrations of substrate (C_12_ or C_14_) at 28 °C in a 1 mL reaction system (50 mM NaH_2_PO_4_, 300 mM NaCl, pH 7.4, 10% glycerol) supplemented with an excess amount of H_2_O_2_ as a cofactor. Aliquots (200 μL) of reactions were removed and quenched at specific time points (usually at 0, 1, 3, and 5 min) by adding 1/10 volume of 1 M HCl. Heptadecanoic acid was then added as the internal standard. Subsequent sample preparation was performed (as above) for GC analysis (as below). Initial rates were calculated from either the substrate consumption for CYP-Aa162 or the 1-alkene production for CYP-Sm46Δ29. Kinetic analyses were performed using OriginPro 8.0 program.

### Gas chromatography (GC) and GC–MS

The GC analytical method for hydrocarbon and fatty acid samples was adapted from Guan et al. [68]. The analyses were performed on an Agilent 7890B gas chromatograph equipped with a capillary column HP-INNOWAX (Agilent Technologies, Santa Clara, CA, USA; cross-linked polyethylene glycerol, i.d. 0.25 μm film thickness, 30 m by 0.25 mm). The helium flow rate was set to 1 mL per min. The oven temperature was controlled initially at 40 °C for 4 min, then increased at the rate of 10 °C min^−1^ to 280 °C, and held for 5 min. The injecting temperature was set to 280 °C with the injection volume of 1 μL under splitless injection conditions. The retention times and signal intensity of fatty acids and alkenes were determined by analyzing and comparing with known authentic fatty acids (C_10_–C_20_), 1-alkenes (C_11_–C_19_), and 1-heptadecanoic acid standards [16]. For GC–MS, the gas chromatography equipment was coupled to an Agilent 5975C MSD single quadrupole mass spectrometer operated under electron ionization mode at 70 eV in the scan range of 50–500 *m/z*. For detection of the hydroxyl fatty acid products, as only authentic standards of α- and β-hydroxy myristic acids were obtained, samples extracted from the C_14_ myristic acid reactions were derivatized with an equal volume of *N*,*O*-bis(trimethylsilyl)trifluoroacetamide (BSTFA) with 1% trimethylchlorosilane at 72 °C for 15 min prior to GC–MS analysis. The GC–MS analysis followed the protocol developed by Rude et al. [13], except for using the Agilent J&W DB-5MS column (i.d. 0.25 μm film thickness, 50 m by 0.25 mm). Peak identity was determined by comparison of retention time and fragmentation pattern with the authentic standard compounds where available and to the National Institute of Standards and Technology, USA mass spectral database. From the reactions using myristic acid as a substrate, we found that the sum of all products (1-tridecene, α- and β-hydroxy myristic acids) almost accurately accounts for at least 99% of the substrate consumption. In addition, as only a minimal level of H_2_O_2_ relative to fatty acid substrate (slightly more than 1:1 molar ratio) was used, no overly oxidized products (such as ketones, di-hydroxyl fatty acids) were observed within the detection limit. Therefore, we quantified the percentage of hydroxylated products for all substrates by subtraction of the 1-alkene production from the total substrate consumption unless otherwise stated. For detection of nonene product from the C_10_ fatty acid reactions, 600 μL of the reaction headspace sample was injected into the GC–MS system using a Hamilton needle syringe. The oven temperature program was as follows: 40 °C for 2 min, then 5 °C per min to 100 °C, and held for 2 min. Quantification of the nonene product was based on the standard curve and reaction controls.

## Additional files

**Additional file 1: Figure S1.** Protein sequence alignment of CYP-Aa162 from *A. acidocaldarius* (GenBank Accession Number: WP_008340313), P450_BSβ_ from *Bacillus subtilis str. 168* (GenBank Accession Number: NP_388092), OleT_JE_ from *Jeotgalicoccus sp.* ATCC 8456 (GenBank Accession Number: ADW41779), CYP-Sm46 (labelled as Sm46 extended) from *Staphylococcus massiliensis* S46 (GenBank Accession Number: EKU50422), CYP-Sm46Δ29 (labelled as Sm46Δ29) from *S. massiliensis* (GenBank Accession Number: WP_039990689) and cytochrome P450 enzymes from other *Staphylococcus* species such as *S. agnetis* (GenBank Accession Number: KFE42911), *S. delphini* (GenBank Accession Number: WP_019165531), *S. intermedius* (GenBank Accession Number: WP_019167377) and *S. pseudintermedius* HKU10-03 (GenBank Accession Number: ADV05454). **Figure S2.** SDS-PAGE showing the purified His-tagged CYP-Aa162 (lane A) and CYP-Sm46Δ29 (lane S). Molecular sizes of the marker bands (lane M), from top to bottom, are 180, 135, 100, 75, 63, 48, 35 and 25 kDa respectively. **Figure S3.** The UV–visible spectra of CYP-Sm46Δ29 (5 μM) under different conditions. (A) The purified CYP-Sm46Δ29 was diluted in 50 mM Na_3_PO_4_ (pH 7.4) buffer containing 300 mM NaCl and 10% glycerol. Spectra are shown for the oxidized ferric form of the enzyme (orange line) and the ferrous-CO complex reduced by the indicated amount of Na_2_S_2_O_4_. (B) The purified CYP-Sm46Δ29 was diluted in 50 mM Na_3_PO_4_ buffer containing 300 mM NaCl and 10% glycerol with different buffer pH as indicated. Then the absorption spectra were recorded respectively for the oxidized ferric form and the ferrous-CO adduct reduced by 10 mM Na_2_S_2_O_4_. The protein precipitates at buffer pH lower than 7.0. (C) A molar excess (600 μM) of C_12_ lauric acid was pre-incubated with the enzyme at room temperature for 5 min before the absorption spectra were recorded. Binding of C_12_ FA did not seem to induce an apparent spin-state transition of the ferric heme. The Soret peak of the C_12_-bound ferrous-CO adduct of the enzyme was still detected at 420 nm. **Figure S4.** UV–visible spectra of the self-sufficient monooxygenase P450_BM3_. The substrate-bound ferric form of P450_BM3_ (solid line) shows a Soret maximum at ~ 416 nm with undistinguishable β-band and a weaker α-band at 570 nm. The reduced ferrous-CO form of P450_BM3_ (dashed line) generated by the subsequent NADPH-initiated electron transfer features a shifted Soret peak to 448 nm. **Figure S5.** Effect of decoupling NADH oxidation and electron transfer on the catalytic conversion of lauric acid (LA) by CYP-Aa162 and CYP-Sm46Δ29. The reactions contained 0.2 mM LA, 2.0 μM CYP-Aa162 (or CYP-Sm46Δ29), 3.0 μM putidaredoxin reductase (PdR), 1 mM NADH in the absence and presence of 1200 U mL^−1^ catalase (Catl.). By subtracting putidaredoxin (Pdx) from the reaction system, the NADH oxidation was mandatorily decoupled from the Class I electron transfer chain to P450 enzymes. Any catalytic activity observed should be supported by the H_2_O_2_ generated from NADH oxidation and O_2_ reduction. The percentage conversion of LA was determined by calculating the substrate consumption based on GC analysis. Results shown are mean ± SD of duplicated experiments. **Figure S6.** The ratios of free fatty acid (FFA) decarboxylation (DC) over hydroxylation (HD) by CYP-Sm46Δ29 against different FFA substrates. The decarboxylation activity was measured by detecting the 1-alkene yield using GC analytical method. The hydroxylation activity was estimated by subtracting the alkene production from the total substrate conversion. This indirect but more convenient method was validated with C_14_ myristic acid substrate by direct measurement of the BSTFA/TMCS derivatized hydroxylation products. Results are shown as mean ± SD of duplicated experiments. **Figure S7.** Phylogenetic tree for CYP-Aa162, CYP-Sm46Δ29 and other CYP152 family members. The sequences were aligned using ClustalW. The Neighbor-joining Tree was generated using MEGA 7.0 package. Bootstrap values shown next to the branches were computed from 1000 bootstrap tests. CYP-Sm46 was found most closely related to the P450 fatty acid decarboxylase OleT_JE_ (CYP152L1), while CYP-Aa162 (CYP152A8) is much closer to the P450 fatty acid hydroxylase P450_BSβ_ (CYP152A1). **Figure S8.** Protein sequence alignment of OleT_JE_, CYP-Sm46Δ29, CYP-Aa162 and P450_BSβ_. *: the only two residues that are distinct in the active sites of these four P450 peroxygenases, which are proposed to be important for product distribution; #: the key catalytic residue. **Figure S9.** Kinetic curves of CYP-Aa162 and CYP-Sm46Δ29 against their optimal fatty acid substrates. (A) C_12_ lauric acid substrate consumption rates by CYP-Aa162 were fitted to Hill equation; (B) 1-undecene formation rates by CYP-Sm46Δ29 were fitted to Hill equation; (C) Solid line: the plot of 1-tridecene formation rates by CYP-Sm46Δ29 as a function of increasing C_14_ myristic acid concentrations, demonstrating substantial substrate inhibition. Dotted line: a hyperbolic curve fitted with Michaelis–Menten equation after truncating the inhibited rates at high C_14_ substrate concentrations. The steady state kinetic parameters were calculated using OriginPro 8.0 and are summarized in Table 2.

**Additional file 2.** The original gene sequence of CYP-Sm46, and the codon-optimized gene sequences of CYP-Sm46 and CYP-Aa162, as well as their corresponding amino acid sequences.

**Additional file 3: Table S1.** Primers used for cloning and site-directed mutagenesis.

## Abbreviations

CYP or P450: cytochrome P450 enzyme
FFA: free fatty acid
LS: low-spin heme iron
CamAB: the P450cam redox partner proteins including putidaredoxin reductase and putidaredoxin
LA: lauric acid
1-UE: 1-undecene
TB: terrific broth
IPTG: isopropyl β-D-1-thiogalactopyranoside
Ni-NTA: nickel-nitrilotriacetic acid resin
GC–MS: gas chromatography–mass spectrometry
DC: decarboxylation
HD: hydroxylation
