# Supplementary material for: In vitro oxidative decarboxylation of free fatty acids to terminal alkenes by two new P450 peroxygenases
Source: Biotechnol Biofuels. 2017 Sep 7;10:208. doi: 10.1186/s13068-017-0894-x (PMC5588734; doi:10.1186/s13068-017-0894-x)
Supplement: Supplementary file 1 — Additional file 1: Figure S1. Protein sequence alignment of CYP-Aa162 from A. acidocaldarius (GenBank Accession Number: WP_008340313), P450BSβ from Bacillus subtilis str. 168 (GenBank Accession Number: NP_388092), OleTJE from Jeotgalicoccus sp. ATCC 8456 (GenBank Accession Number: ADW41779), CYP-Sm46 (labelled as Sm46 extended) from Staphylococcus massiliensis S46 (GenBank Accession Number: EKU50422), CYP-Sm46Δ29 (labelled as Sm46Δ29) from S. massiliensis (GenBank Accession Number: WP_039990689) and cytochrome P450 enzymes from other Staphylococcus species such as S. agnetis (GenBank Accession Number: KFE42911), S. delphini (GenBank Accession Number: WP_019165531), S. intermedius (GenBank Accession Number: WP_019167377) and S. pseudintermedius HKU10-03 (GenBank Accession Number: ADV05454). Figure S2. SDS-PAGE showing the purified His-tagged CYP-Aa162 (lane A) and CYP-Sm46Δ29 (lane S). Molecular sizes of the marker bands (lane M), from top to bottom, are 180, 135, 100, 75, 63, 48, 35 and 25 kDa respectively. Figure S3. The UV–visible spectra of CYP-Sm46Δ29 (5 μM) under different conditions. (A) The purified CYP-Sm46Δ29 was diluted in 50 mM Na3PO4 (pH 7.4) buffer containing 300 mM NaCl and 10% glycerol. Spectra are shown for the oxidized ferric form of the enzyme (orange line) and the ferrous-CO complex reduced by the indicated amount of Na2S2O4. (B) The purified CYP-Sm46Δ29 was diluted in 50 mM Na3PO4 buffer containing 300 mM NaCl and 10% glycerol with different buffer pH as indicated. Then the absorption spectra were recorded respectively for the oxidized ferric form and the ferrous-CO adduct reduced by 10 mM Na2S2O4. The protein precipitates at buffer pH lower than 7.0. (C) A molar excess (600 μM) of C12 lauric acid was pre-incubated with the enzyme at room temperature for 5 min before the absorption spectra were recorded. Binding of C12 FA did not seem to induce an apparent spin-state transition of the ferric heme. The Soret peak of the C12-bound ferrous-CO adduct [file 13068_2017_894_MOESM1_ESM.pdf]

```
A. acidocaldarius     ............................MNQCIPRDRTFDSSLALIKEGYLFIKNRVDQY    32
B. subtilis           ............................MNEQIPHDKSLDNSLTLLKEGYLFIKNRTERY    32
OleTJE                .............................MATLKRDKGLDNTLKVLKQGYLYTTNQRNRL    31
S. agnetis            ............................MAKQLPKDPGLDNTFKVLKEAYTYVPCRLEKF    32
S. delphini           ............................MAKKLPKDTGLDNTLKIINEAYTYVPKRLEKF    32
S. intermedius        ............................MAKKLPKDTGLDNTFKIINEAYTYVPKRLEQF    32
S. pseudintermedius   ............................MAKKLPKDTGLDNTLKMINEAYTYVPKRLEKF    32
Sm46Δ29               ............................MAKKLPKVKGLDNTVDIIKGGYTYVPGKLEEF    32
Sm46 extended         MFVDSILVLRLNLLKTGIQLEMKNGGIKVAKKLPKVKGLDNTVDIIKGGYTYVPGKLEEF    60

A. acidocaldarius     QSDIFEARLLL.ENVVCMHGAEAAKLFYNTELFQRQGALPKRVQKTLFGENAIQTLDGTA    91
B. subtilis           NSDLFQARLLG.KNFICMTGAEAAKVFYDTDRFQRQNALPKRVQKSLFGVNAIQGMDGSA    91
OleTJE                NTSVFQTKALGGKPFVVVTGKEGAEMFYNNDVVQREGMLPKRIVNTLFGKGAIHTVDGKK    91
S. agnetis            NSKAFQTTGMGMKPIAVISGKEAAELFYNNDVMQREKTLPKRVVNTLFGKGAIHTTKGKV    92
S. delphini           GTKAFETRALGMKPVVVISGKAAAELFYDNDKISRKGTLPKRIVHTLFGKGAIHTTEGKV    92
S. intermedius        GTKAFETRALGMKPVVVISGKAAAKLFYDNDKISRKGTLPKRIVHTLFGKGAIHTTEGKV    92
S. pseudintermedius   GTKAFETRALGMKPIVVISGKAAAELFYDNDKISRKGTLPKRIVHTLFGKGAIHTTEGKV    92
Sm46Δ29               DSKAFEVRALGGKKIAVMSGKEAAEIFYDNEKMERQGTLPKRIVNTLFGKGAIHTTAGKK    92
Sm46 extended         DSKAFEVRALGGKKIAVMSGKEAAEIFYDNEKMERQGTLPKRIVNTLFGKGAIHTTAGKK   120

A. acidocaldarius     HLHRKQLFLSLLTPDQEKSLATLATTQWRECAKVWENADRVVLFEEAKRMLCRIACQWTG   151
B. subtilis           HIHRKMLFLSLMTPPHQKRLAELMTEEWKAAVTRWEKADEVVLFEEAKEILCRVACYWAG   151
OleTJE                HVDRKALFMSLMTEGNLNYVRELTRTLWHANTQRMESMDEVNIYRESIVLLTKVGTRWAG   151
S. agnetis            HVDRKALFMSLMTEENLKYLRELTRNHWFMHTEHMQNQKEVNIYKESIYVLTKIGFRWAG   152
S. delphini           HVDRKALFMSLMTEKNLKYLRELTRNYWFMHTERMQNKDEVNVYQEAGLILTKVGFRWAG   152
S. intermedius        HVDRKALFMSLMTEKNLKYLRELTRNYWFMHTERMQNMDEVNVYKEAGLILTQVGFRWAG   152
S. pseudintermedius   HVDRKALFMSLMTEENLKYLRELTRNYWFMHTERMQNKDEVNVYQEAGLILTKVGFRWAG   152
Sm46Δ29               HVDRKALFMSLMTDENLNYLRELTRNYWFMNTERMQSMDKVNVYNESIYMLTKIGFRWAG   152
Sm46 extended         HVDRKALFMSLMTDENLNYLRELTRNYWFMNTERMQSMDKVNVYNESIYMLTKIGFRWAG   180

A. acidocaldarius     VPLDESEVSKRADDFGAMVDAFGAVGPRHWKG.....RRARARAEAWLRQMIDEIRIGLR   206
B. subtilis           VPLKETEVKERADDFIDMVDAFGAVGPRHWKG.....RRARPRAEEWIEVMIEDARAGLL   206
OleTJE                VQAPPEDIERIATDMDIMIDSFRALG.GAFKG.YKASKEARRRVEDWLEEQIIETRKGNI   209
S. agnetis            IHQTTEEAEQNAKDMDIMIDSFKGLG.QTIGGYRKAKKARARVEQFLEKQIIAVRKGKI   211
S. delphini           LKQTDEQAAQNAEDMNTMIDSFSGLG.QSLKG.YREAKKARARVEQFLQDQIEAVRAGQQ   210
S. intermedius        LKQTDEEAAKNAEDMNIMIDSFSGLG.QSLKG.YRQAKKARARVEQFLQDQIEAVRAGKQ   210
S. pseudintermedius   LKQTDEQAAQNAEDMNTMIDSFSGLG.QSLKG.YREAKKARARVEQFLQEQIEAVRVGQQ   210
Sm46Δ29               IIQTPEEAEQNAKDMDTMINSFVSLG.SAYKG.YKKAKKARKRVEDFLEKQIIDVRKGKL   210
Sm46 extended         IIQTPEEAEQNAKDMDTMINSFVSLG.SAYKG.YKKAKKARKRVEDFLEKQIIDVRKGKL   238

A. acidocaldarius     SVDEHTPLHVVAFWRDVNGNLLDAQMVAIELINLLRPIVAISTFITFSALALHEHPTWRD   266
B. subtilis           KTTSGTALHEMAFHTQEDGSQLDSRMAAIELINVLRPIVAISYFLVFSALALHEHPKYKE   266
OleTJE                HPPEGTALYEFAHWEDYLGNPMDSRTCAIDLMNTFRPLIAINRFVSFGLHAMNENPITRE   269
S. agnetis            NAEPGTALYEFAHWEDYKGNPMDARLCAIDLMNVVRPLAAVNRFVSYAVKAMIEYDQERI   271
S. delphini           YAEPGTALYEFAHWKDLNDQPMDPHLCAVDLMNIVRPLVAVNRFVSYGVKALIEFDQERK   270
S. intermedius        YAEPGTALYEFAHWKDLNNQPMDSHLCAVDLMNIVRPLVAVNRFVSYGVKALIEFDQERK   270
S. pseudintermedius   YAEPGTALYEFAHWKDLNDQPMDPHLCAVDLMNIVRPLVAVNRFVSYGVKALIEFDQERK   270
Sm46Δ29               HPEEGTALYEFAHWEDLNDNPMDSHLCAVDLMNVVRPLAAINRFISYGVKVLIEFDQEKE   270
Sm46 extended         HPEEGTALYEFAHWEDLNDNPMDSHLCAVDLMNVVRPLAAINRFISYGVKVLIEFDQEKE   298

A. acidocaldarius     RLKARNEADIE.MFVQEVRRYYPFAPFLGARVKKDFVWRGYEFKRGTLVLLDVYGTHHDA   325
B. subtilis           WLRSGNSRERE.MFVQEVRRYYPFGPFLGALVKKDFVWNNCEFKKGTSVLLDLYGTNHDP   325
OleTJE                KIKS..EPDYAYKFAQEVRRYYPFVPFLPGKAKVDIDFQGVTIPAGVGLALDVYGTTHDE   327
S. agnetis            KLQVSDDPNYAYKFAQEVRRIFPFVPFLPGKLKKNIEFDGYRIKKGTFTLLDVFGTTHDP   331
S. delphini           KLQVTHDPNYAYKFAQEVRRIFPFVPFLPGRLKQTVEFDGFKLKKGTLTVLDIFGTTHDP   330
S. intermedius        KLQVTNDPNYAYKFAQEVRRIFPFVPFLPGRLTKTVEFDGFKIKKGTLVALDIFGTTHDP   330
S. pseudintermedius   KLQVTNDPNYAYKFAQEVRRIFPFVPFLPGRLKKTVEFDGFKLKKGTLTVLDIFGTTHDP   330
Sm46Δ29               KLRLENNEDYAYKFAQEVRRIFPFVPYLPGRAAVDLEYDGYKIPAGMMTALDVYGTTHDE   330
Sm46 extended         KLRLENNEDYAYKFAQEVRRIFPFVPYLPGRAAVDLEYDGYKIPAGMMTALDVYGTTHDE   358

A. acidocaldarius     RLWDSPNEFRPERFMRKTVGPFDLIPQGGGDSHTGHRCPGEGATIEIMKASVDFLVNQID   385
B. subtilis           RLWDHPDEFRPERFAEREENLFDMIPQGGGHAEKGHRCPGEGITIEVMKASLDFLVHQIE   385
OleTJE                SLWDDPNEFRPERFETWDGSPFDLIPQGGGDYWTNHRCAGEWITVIIMEETMKYFAEKIT   387
S. agnetis            ELFENPYQFNPDRFENWDGSPFDLIPQGGGDFYTNHRCAGEWMTIIVMEETIKYFANKID   391
S. delphini           ELFENPYQFNPDRFDNWDGSPFDLIPQGGGDFYTNHRCAGEWMTVIVMEETIQYFANKID   390
S. intermedius        ELFENPYQFNPDRFENWDGSPFDLIPQGGGDFYTNHRCAGEWMTVIVMEETIQYFANKID   390
S. pseudintermedius   ELFENPYQFNPDRFDNWDGSPFDLIPQGGGDFYTNHRCAGEWMTVIVMEETIQYFANKID   390
Sm46Δ29               DLWENPDQFNPNRFDNWDGSPFDLIPQGGGDFYTNHRCAGEWITVIIMEETMKYFANKIE   390
Sm46 extended         DLWENPDQFNPNRFDNWDGSPFDLIPQGGGDFYTNHRCAGEWITVIIMEETMKYFANKIE   418

A. acidocaldarius     FEVPAQDLSYRLDVMPTLPKSGFVLTHVHRKFIASPTIATPNGSEALPSE            435
B. subtilis           YDVPEQSLHYSLARMPSLPESGFVMSGIRRKS..................            417
OleTJE                YDVPEQDLEVDLNSIPGYVKSGFVIKNVREVVDRT...............            422
S. agnetis            FEAPSQDLSVKLDQFPGKVTSGTIIQNVRPRVQR................            425
S. delphini           FEAPAQDLSVKLDQFPGKVTSGTIIKSVYPRI..................            422
S. intermedius        FDAPAQDLSVKLDQFPGKVTSGTVIKNVYPRI..................            422
S. pseudintermedius   FVVPAQDLSVKLSQFPGKVTSGTMIKNVYPRI..................            422
Sm46Δ29               FDVPSQDLSVKLDKLPGNVTSGTIISNVRPRVARK...............            425
Sm46 extended         FDVPSQDLSVKLDKLPGNVTSGTIISNVRPRVARK...............            453
```

Figure S1. Protein sequence alignment of CYP-Aa162 from *A. acidocaldarius* (GenBank accession number: WP_008340313), $P450_{BS\beta}$ from *Bacillus subtilis str. 168* (GenBank accession number: NP_388092), $OleT_{JE}$ from *Jeotgalicoccus sp.* ATCC 8456 (GenBank accession number: ADW41779), CYP-Sm46 (labelled as Sm46 extended) from *Staphylococcus massiliensis* S46 (GenBank accession number: EKU50422), CYP-Sm46Δ29 (labelled as Sm46Δ29) from *S. massiliensis* (GenBank accession number: WP_039990689) and cytochrome P450 enzymes from other *Staphylococcus* species such as *S. agnetis* (GenBank accession number: KFE42911), *S. delphini* (GenBank accession number: WP_019165531), *S. intermedius* (GenBank accession number: WP_019167377) and *S. pseudintermedius* HKU10-03 (GenBank accession number: ADV05454).

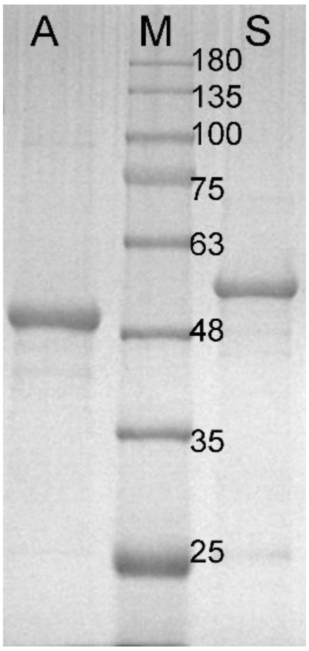

Figure S2. SDS-PAGE showing the purified His-tagged CYP-Aa162 (lane A) and CYP-Sm46Δ29 (lane S). Molecular sizes of the marker bands (lane M), from top to bottom, are 180, 135, 100, 75, 63, 48, 35 and 25 kDa respectively.

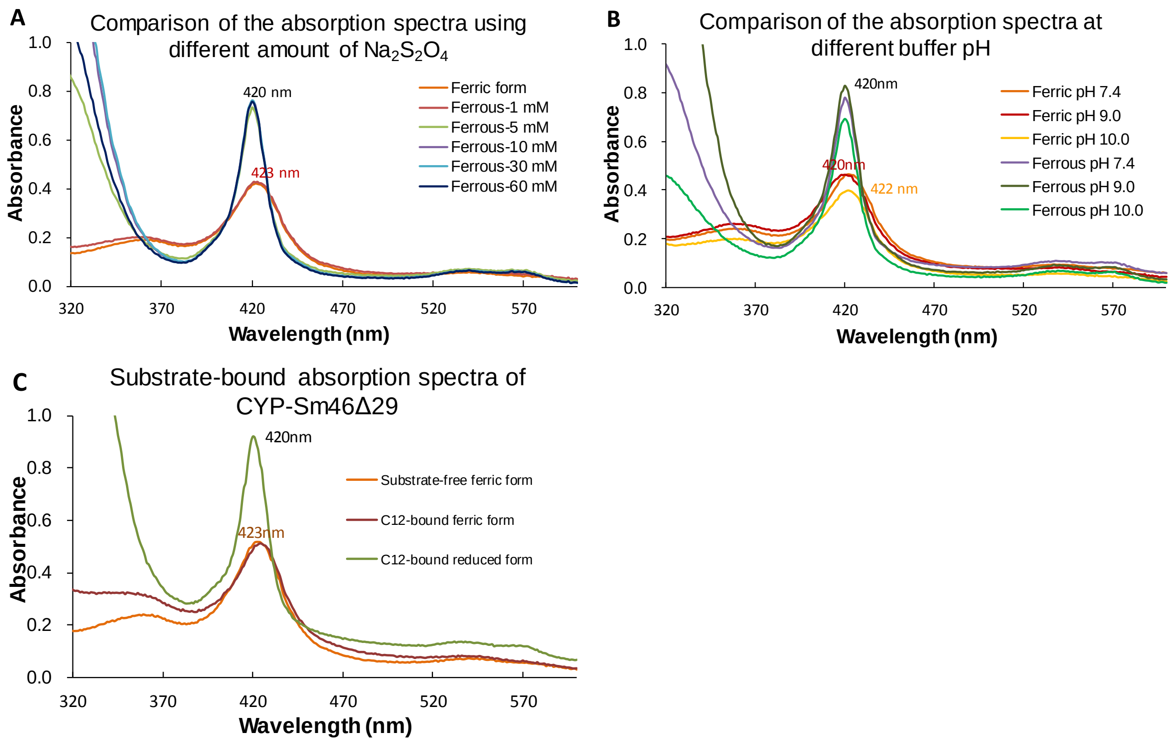

Figure S3. The UV-visible spectra of CYP-Sm46Δ29 (5 μM) under different conditions. (A) The purified CYP-Sm46Δ29 was diluted in 50 mM Na_3PO_4 (pH 7.4) buffer containing 300 mM NaCl and 10% glycerol. Spectra are shown for the oxidized ferric form of the enzyme (orange line) and the ferrous-CO complex reduced by the indicated amount of $Na_2S_2O_4$. (B) The purified CYP-Sm46Δ29 was diluted in 50 mM Na_3PO_4 buffer containing 300 mM NaCl and 10% glycerol with different buffer pH as indicated. Then the absorption spectra were recorded respectively for the oxidized ferric form and the ferrous-CO adduct reduced by 10 mM $Na_2S_2O_4$. The protein precipitates at buffer pH lower than 7.0. (C) A molar excess (600 μM) of C_{12} lauric acid was pre-incubated with the enzyme at room temperature for 5 min before the absorption spectra were recorded. Binding of C_{12} FA did not seem to induce an apparent spin-state transition of the ferric heme. The Soret peak of the C_{12}-bound ferrous-CO adduct of the enzyme was still detected at 420 nm.

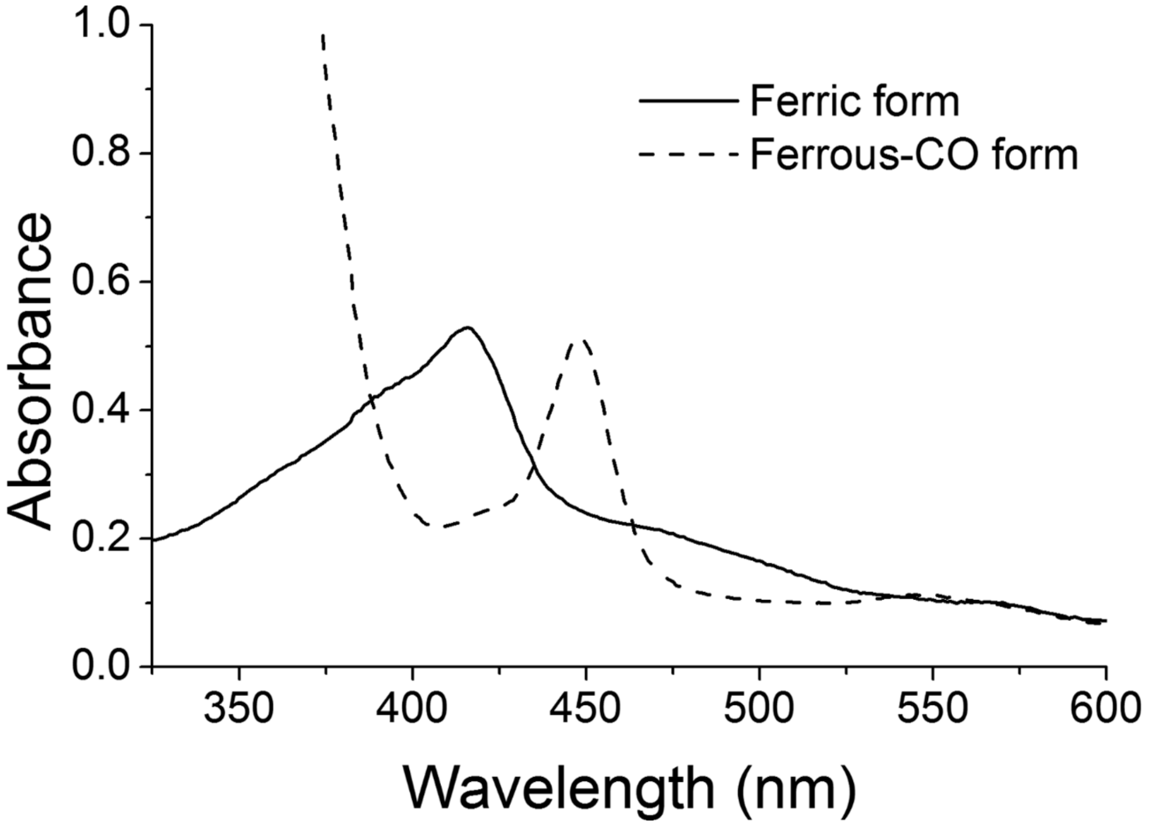

Figure S4. UV-visible spectra of the self-sufficient monooxygenase $P450_{BM3}$. The substrate-bound ferric form of $P450_{BM3}$ (solid line) shows a Soret maximum at ~ 416 nm with undistinguishable *β*-band and a weaker *α*-band at 570 nm. The reduced ferrous-CO form of $P450_{BM3}$ (dashed line) generated by the subsequent NADPH-initiated electron transfer features a shifted Soret peak to 448 nm.

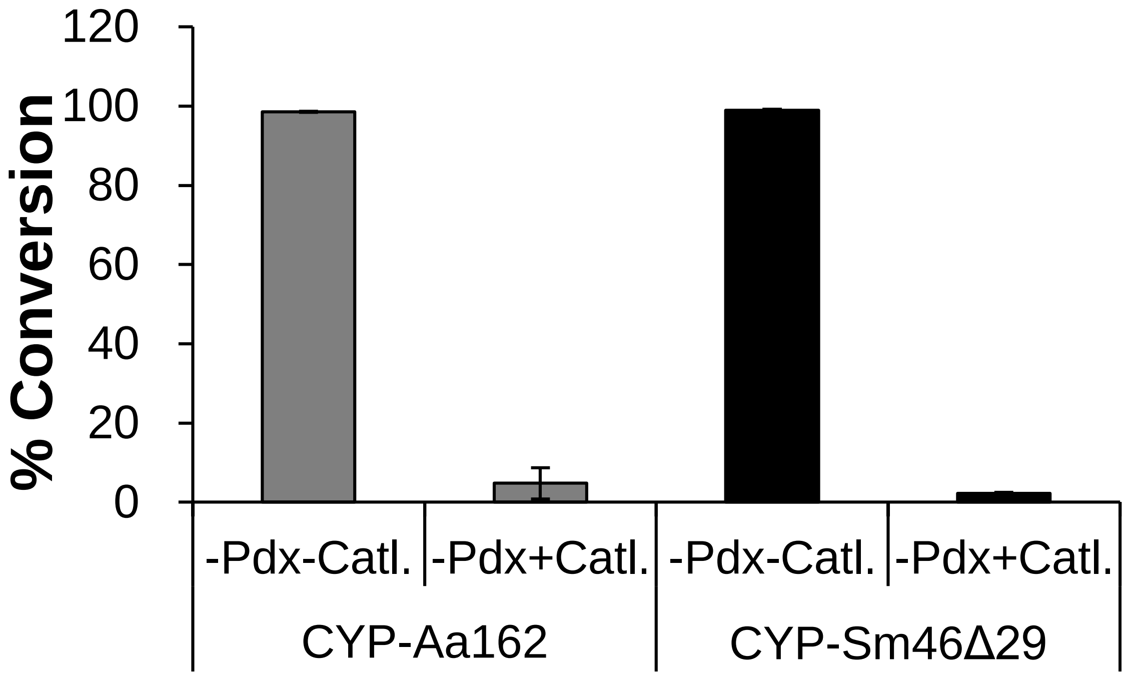

Figure S5. Effect of decoupling NADH oxidation and electron transfer on the catalytic conversion of lauric acid (LA) by CYP-Aa162 and CYP-Sm46Δ29. The reactions contained 0.2 mM LA, 2.0 μM CYP-Aa162 (or CYP-Sm46Δ29), 3.0 μM putidaredoxin reductase (PdR), 1 mM NADH in the absence and presence of 1200 U/ml catalase (Catl.). By subtracting putidaredoxin (Pdx) from the reaction system, the NADH oxidation was mandatorily decoupled from the Class I electron transfer chain to P450 enzymes. Any catalytic activity observed should be supported by the H_2O_2 generated from NADH oxidation and O_2 reduction. The percentage conversion of LA was determined by calculating the substrate consumption based on GC analysis. Results shown are mean ± SD of duplicated experiments.

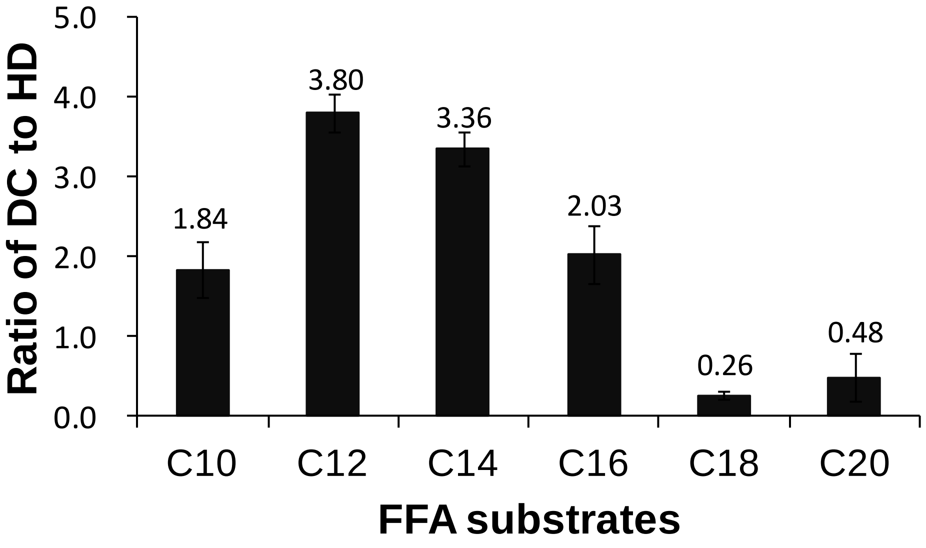

Figure S6. The ratios of free fatty acid (FFA) decarboxylation (DC) over hydroxylation (HD) by CYP-Sm46Δ29 against different FFA substrates. The decarboxylation activity was measured by detecting the 1-alkene yield using GC analytical method. The hydroxylation activity was estimated by subtracting the alkene production from the total substrate conversion. This indirect but more convenient method was validated with C_{14} myristic acid substrate by direct measurement of the BSTFA/TMCS derivatized hydroxylation products. Results are shown as mean ± SD of duplicated experiments.

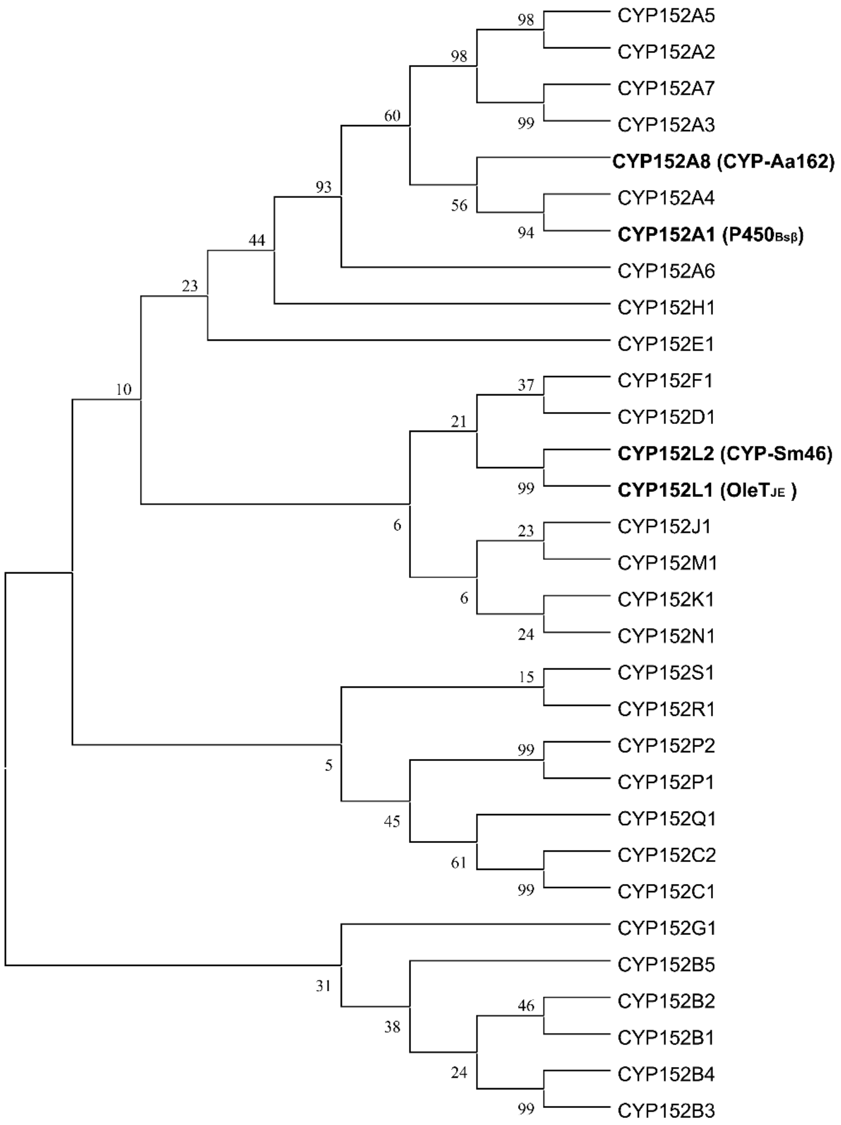

Figure S7. Phylogenetic tree for CYP-Aa162, CYP-Sm46Δ29 and other CYP152 family members. The sequences were aligned using Clustal W. The Neighbor-joining Tree was generated using MEGA 7.0 package. Bootstrap values shown next to the branches were computed from 1000 bootstrap tests. CYP-Sm46 is most closely related to the P450 fatty acid decarboxylase $OleT_{JE}$ (CYP152L1), while CYP-Aa162 (CYP152A8) is much closer to the P450 fatty acid hydroxylase $P450_{BS\beta}$ (CYP152A1).

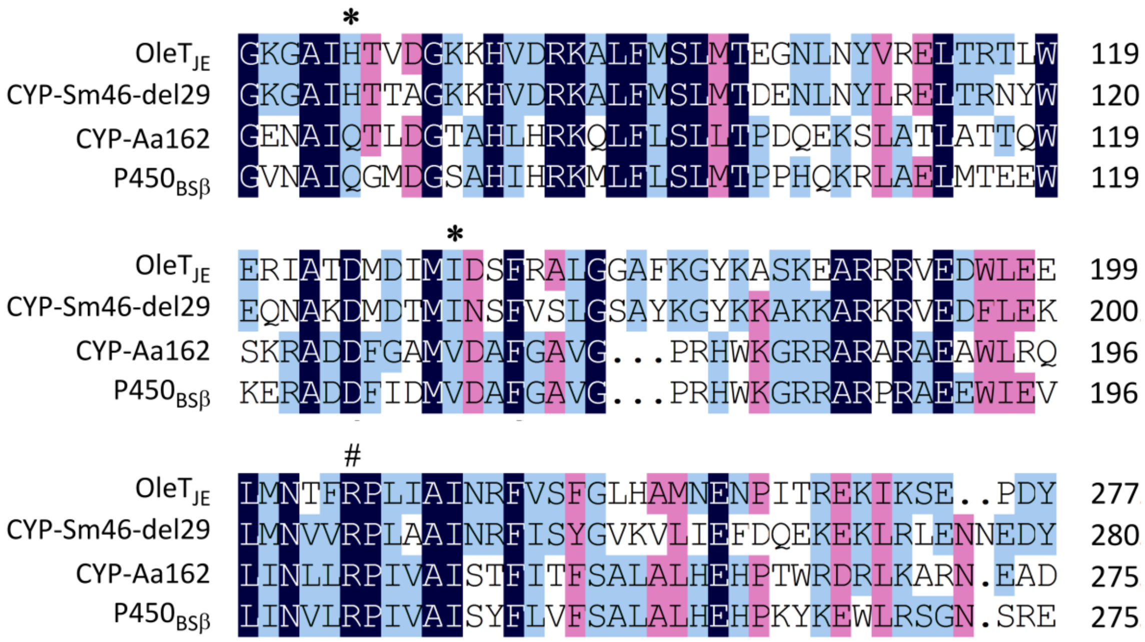

Figure S8. Protein sequence alignment of $OleT_{JE}$, CYP-Sm46Δ29, CYP-Aa162 and $P450_{BS\beta}$. *: the only two residues that are distinct in the active sites of these four P450 peroxygenases, which are proposed to be important for product distribution; #: the key catalytic residue.

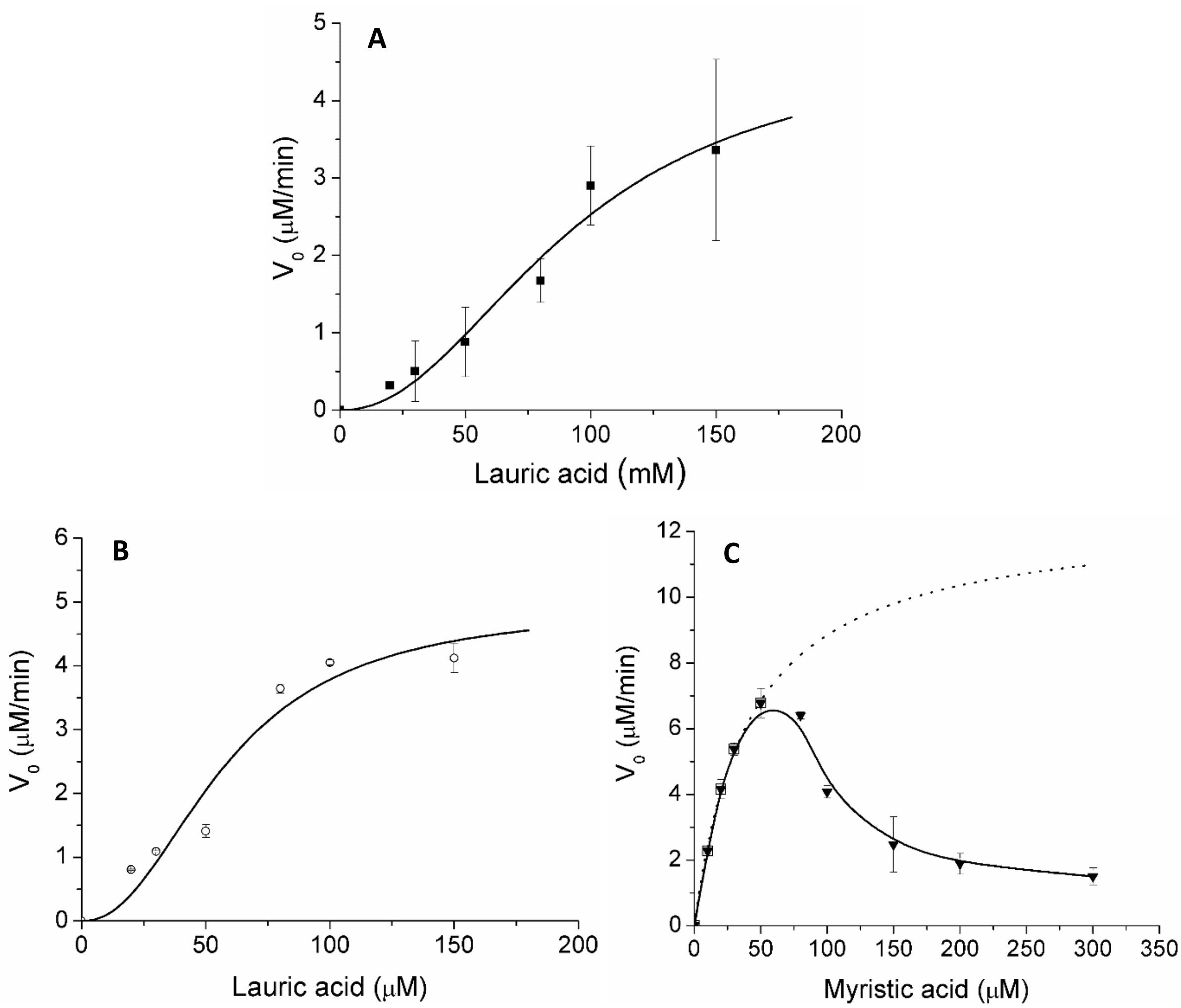

Figure S9. Kinetic curves of CYP-Aa162 and CYP-Sm46Δ29 against their optimal fatty acid substrates. (A) C_{12} lauric acid substrate consumption rates by CYP-Aa162 were fitted to Hill equation; (B) 1-undecene formation rates by CYP-Sm46Δ29 were fitted to Hill equation; (C) Solid line: the plot of 1-tridecene formation rates by CYP-Sm46Δ29 as a function of increasing C_{14} myristic acid concentrations, demonstrating substantial substrate inhibition. Dotted line: a hyperbolic curve fitted with Michaelis-Menten equation after truncating the inhibited rates at high C_{14} substrate concentrations. The steady state kinetic parameters were calculated using OriginPro 8.0 and are summarized in Table 2.
