# Supplementary material for: In vitro oxidative decarboxylation of free fatty acids to terminal alkenes by two new P450 peroxygenases
Source: Biotechnol Biofuels. 2017 Sep 7;10:208. doi: 10.1186/s13068-017-0894-x (PMC5588734; doi:10.1186/s13068-017-0894-x)
Supplement: Supplementary file 2 — Additional file 2. The original gene sequence of CYP-Sm46, and the codon-optimized gene sequences of CYP-Sm46 and CYP-Aa162, as well as their corresponding amino acid sequences. [file 13068_2017_894_MOESM2_ESM.docx]

The original DNA sequence of CYP-Sm46 gene including the initiation region. The coding strand equivalent to the mRNA sequence is shown. The two possible start codons are labeled in red. The possible Shine-Dalgarno sites are shown in bold and underlined.

AAAGACGCACTAATCCGCCATGGTTCTATAAAAAATCGTTGTGAGCATCAATTTATTATTACAGATTTAAACTCTATGTTACGATT**AGGATG**AAATGTTTGTAGATTCGATACTTGTGTTAAGATTAAATTTATTAAAAACGGGTATACAATTAGAAATGAAAAA**TGGGGG**AATCAAAGTGGCAAAGAAACTACCTAAGGTTAAAGGCCTAGATAACACAGTAGACATTATTAAAGGCGGGTATACATACGTACCTGGCAAATTAGAAGAATTTGATTCTAAAGCATTTGAAGTACGCGCATTAGGCGGTAAGAAAATTGCTGTTATGAGCGGTAAAGAAGCGGCAGAAATTTTCTATGATAATGAAAAAATGGAAAGACAAGGTACTTTACCAAAACGTATCGTAAACACTTTATTTGGTAAAGGTGCAATTCATACAACTGCTGGTAAGAAGCACGTTGACCGTAAAGCTTTATTTATGTCACTTATGACAGATGAAAATCTTAACTACTTACGTGAATTAACACGTAATTATTGGTTCATGAATACTGAACGTATGCAAAGCATGGATAAAGTTAACGTATATAACGAATCAATTTATATGTTAACTAAAATCGGCTTCCGTTGGGCTGGTATCATCCAAACGCCTGAAGAAGCAGAACAAAATGCGAAAGACATGGATACTATGATTAACTCATTCGTATCTTTAGGTTCAGCTTACAAAGGTTATAAGAAAGCTAAAAAAGCACGTAAACGTGTTGAAGATTTCTTAGAAAAACAAATTATCGATGTGCGTAAAGGTAAATTACACCCTGAAGAAGGTACTGCGTTATACGAATTCGCGCATTGGGAAGATTTAAACGATAACCCAATGGATTCTCACTTATGTGCAGTAGACTTAATGAACGTTGTGCGCCCATTAGCTGCAATCAACCGTTTCATCAGCTATGGTGTTAAAGTATTAATCGAATTCGATCAAGAAAAAGAAAAATTACGTCTTGAAAATAATGAAGACTATGCGTATAAATTCGCTCAAGAAGTACGTCGTATCTTCCCATTCGTACCATACTTACCAGGTAGAGCAGCTGTTGATTTAGAATATGACGGCTACAAAATCCCTGCAGGTATGATGACAGCATTAGATGTTTATGGTACGACACATGATGAAGATTTATGGGAAAACCCAGACCAATTCAATCCTAACCGTTTTGATAACTGGGACGGTAGCCCATTCGACTTAATTCCACAAGGTGGCGGTGACTTCTATACGAACCACAGATGTGCTGGTGAGTGGATCACAGTTATCATTATGGAAGAAACAATGAAATATTTCGCGAATAAGATTGAATTTGATGTACCGTCTCAAGATTTATCAGTTAAGCTTGATAAATTACCAGGTAACGTAACAAGCGGTACAATCATTAGTAATGTACGTCCACGTGTTGCGCGTAAATAA

Codon-optimized gene sequence of CYP-Sm46. *Nde*I and *Xho*I restriction sites are underlined. Start and stop codons are shown in bold. Sequence deleted in *CYP-Sm46-Δ29* is highlighted in red.

CAT**ATG**TTCGTGGATAGCATTCTGGTTCTGCGCCTGAACCTGCTGAAGACAGGCATCCAGCTGGAGATGAAGAACGGTGGCATCAAA**GTG**GCAAAAAAGCTGCCTAAAGTGAAAGGTCTGGACAACACCGTGGACATCATCAAGGGTGGCTATACCTACGTGCCTGGCAAACTGGAGGAGTTCGACAGCAAAGCATTCGAAGTGCGCGCCCTGGGTGGCAAGAAGATCGCAGTGATGAGCGGCAAGGAAGCCGCCGAGATTTTTTATGATAACGAAAAAATGGAGCGTCAGGGTACCCTGCCGAAGCGCATCGTGAACACACTGTTCGGTAAAGGCGCCATTCATACCACCGCCGGCAAGAAACATGTGGATCGCAAGGCACTGTTCATGAGTCTGATGACCGATGAAAATTTAAATTATCTGCGCGAACTGACACGCAACTATTGGTTTATGAATACAGAACGCATGCAGAGCATGGATAAAGTGAATGTGTACAATGAAAGCATTTATATGCTGACCAAAATTGGCTTCCGCTGGGCCGGTATCATTCAGACCCCTGAAGAGGCCGAGCAGAATGCCAAAGACATGGACACCATGATCAACAGCTTTGTGAGCCTGGGCAGCGCCTACAAGGGTTACAAAAAAGCCAAGAAAGCCCGCAAGCGCGTGGAAGATTTTCTGGAGAAACAAATTATCGACGTTCGTAAAGGCAAACTGCATCCGGAGGAAGGTACCGCCCTGTACGAATTCGCCCATTGGGAAGACCTGAACGATAACCCGATGGACAGCCATCTGTGCGCCGTTGATCTGATGAACGTTGTTCGCCCGCTGGCAGCAATTAACCGCTTCATTAGCTACGGCGTTAAAGTGCTGATCGAATTCGACCAGGAAAAAGAAAAGCTGCGCCTGGAGAACAACGAGGACTACGCCTACAAGTTCGCACAGGAAGTGCGCCGTATCTTTCCGTTCGTGCCTTACTTACCGGGTCGCGCCGCCGTGGATCTGGAGTATGATGGCTATAAGATCCCGGCCGGTATGATGACCGCCCTGGATGTTTACGGTACCACACACGATGAGGATCTGTGGGAGAATCCGGATCAGTTCAACCCGAATCGTTTTGATAACTGGGACGGCAGTCCGTTTGATCTGATTCCGCAGGGCGGTGGCGATTTCTACACCAATCATCGTTGCGCCGGCGAGTGGATCACCGTGATTATTATGGAAGAAACAATGAAATACTTTGCCAACAAAATTGAATTCGATGTGCCGAGTCAGGACCTGAGCGTTAAACTGGACAAACTGCCTGGCAACGTGACCAGCGGTACCATCATTAGCAACGTGCGTCCGCGTGTTGCCCGCAAA**TAA**CTCGAG

Codon-optimized gene sequence of CYP-Aa162. *Nde*I and *Xho*I restriction sites are underlined. Start and stop codons are shown in bold.

CAT**ATG**AATCAGTGCATTCCGCGCGATCGCACCTTTGATAGCAGTCTGGCCCTGATCAAAGAGGGCTACCTGTTCATCAAGAATCGCGTTGACCAGTACCAGAGCGATATCTTTGAAGCCCGCCTGCTGCTGGAAAATGTGGTGTGCATGCACGGCGCCGAAGCAGCCAAGCTGTTTTACAATACCGAACTGTTTCAACGTCAGGGCGCATTACCGAAGCGTGTGCAGAAAACACTGTTCGGCGAGAACGCCATTCAGACCCTGGATGGTACCGCACATCTGCATCGCAAACAGCTGTTCCTGAGTCTGCTGACACCGGACCAGGAAAAAAGCCTGGCAACCCTGGCCACCACCCAATGGCGTGAATGTGCCAAGGTGTGGGAAAATGCCGATCGTGTGGTGCTGTTTGAAGAGGCCAAACGCATGCTGTGCCGTATTGCCTGCCAGTGGACCGGCGTTCCGTTAGATGAAAGCGAAGTGAGCAAACGCGCCGATGACTTTGGTGCCATGGTGGATGCCTTTGGTGCAGTTGGCCCGCGTCATTGGAAAGGCCGCCGTGCACGTGCACGTGCAGAAGCATGGCTGCGTCAAATGATCGACGAGATTCGCATCGGCCTGCGTAGCGTTGATGAACACACCCCGCTGCATGTGGTGGCCTTTTGGCGCGACGTGAACGGCAATCTGTTAGATGCCCAGATGGTGGCCATCGAACTGATTAATCTGCTGCGTCCGATCGTGGCCATTAGCACCTTCATCACCTTCAGCGCCCTGGCCCTGCATGAGCATCCGACATGGCGCGATCGTCTGAAAGCACGCAATGAGGCCGACATCGAGATGTTTGTGCAAGAAGTTCGCCGTTATTACCCGTTCGCCCCTTTTCTGGGTGCCCGCGTGAAGAAAGACTTTGTGTGGCGCGGTTATGAATTTAAACGCGGCACCCTGGTGCTGCTGGATGTTTACGGCACCCACCATGATGCCCGCCTGTGGGACAGCCCGAATGAATTTCGCCCGGAACGCTTTATGCGCAAAACCGTGGGCCCGTTTGACCTGATCCCTCAGGGTGGTGGTGACAGCCATACAGGTCACCGCTGCCCGGGCGAAGGTGCAACCATCGAGATCATGAAAGCCAGCGTTGATTTCCTGGTGAACCAGATCGACTTTGAAGTTCCTGCCCAGGATCTGAGCTATCGCCTGGATGTTATGCCGACCCTGCCGAAAAGTGGCTTTGTGCTGACCCATGTGCACCGTAAGTTCATTGCCAGCCCGACCATTGCCACCCCGAATGGTAGCGAGGCACTGCCTAGTGAGGTG**TAA**CTCGAG

# Amino acid sequences of CYP-Aa162 and CYP-Sm46Δ29

1. CYP-Aa162 (WP_008340313):

mnqciprdrtfdsslalikegylfiknrvdqyqsdifearlllenvvcmhgaeaaklfyntelfqrqgalpkrvqktlfgenaiqtldgtahlhrkqlflslltpdqekslatlattqwrecakvwenadrvvlfeeakrmlcriacqwtgvpldesevskraddfgamvdafgavgprhwkgrrararaeawlrqmideiriglrsvdehtplhvvafwrdvngnlldaqmvaielinllrpivaistfitfsalalhehptwrdrlkarneadiemfvqevrryypfapflgarvkkdfvwrgyefkrgtlvlldvygthhdarlwdspnefrperfmrktvgpfdlipqgggdshtghrcpgegatieimkasvdflvnqidfevpaqdlsyrldvmptlpksgfvlthvhrkfiasptiatpngsealpsev

1. CYP-Sm46Δ29 (WP_039990689):

Makklpkvkgldntvdiikggytyvpgkleefdskafevralggkkiavmsgkeaaeifydnekmerqgtlpkrivntlfgkgaihttagkkhvdrkalfmslmtdenlnylreltrnywfmntermqsmdkvnvynesiymltkigfrwagiiqtpeeaeqnakdmdtminsfvslgsaykgykkakkarkrvedflekqiidvrkgklhpeegtalyefahwedlndnpmdshlcavdlmnvvrplaainrfisygvkvliefdqekeklrlennedyaykfaqevrrifpfvpylpgraavdleydgykipagmmtaldvygtthdedlwenpdqfnpnrfdnwdgspfdlipqgggdfytnhrcagewitviimeetmkyfankiefdvpsqdlsvkldklpgnvtsgtiisnvrprvark
