# Supplementary material for: In vitro oxidative decarboxylation of free fatty acids to terminal alkenes by two new P450 peroxygenases
Source: Biotechnol Biofuels. 2017 Sep 7;10:208. doi: 10.1186/s13068-017-0894-x (PMC5588734; doi:10.1186/s13068-017-0894-x)
Supplement: Supplementary file 3 — Additional file 3: Table S1. Primers used for cloning and site-directed mutagenesis. [file 13068_2017_894_MOESM3_ESM.docx]

Table S1. **Primers used for cloning and site-directed mutagenesis**

| Primer names | Sequences 5→3 | |
| --- | --- | --- |
| Sm46-Δ29-F | GTCCATATGGCAAAAAAGCTGCCTAAAGTG |  |
| Sm46-Δ29-R | GTACTCGAGTTATTTGCGGGCAACACGCGG |  |
| Aa-85-170-F | GGAATTCCATATGAATCAGTGCATTCCGCGCG |  |
| Aa-85-F | CACTGTTCGGCGAGAACGCCATT**CAC**ACCCTGGATGGTACCGCACATC |  |
| Aa-85-R | GCAGATGTGCGGTACCATCCAGGGT**GTG**AATGGCGTTCTCGCCGAAC |  |
| Aa-170-F | CGCGCCGATGACTTTGGTGCCATG**ATC**GATGCCTTTGGTGCAGTTGGC |  |
| Aa-170-R | GCGGGCCAACTGCACCAAAGGCATC**GAT**CATGGCACCAAAGTCATCGGCG |  |
| Aa-85-170-R | GCCGCTCGAGTTACACCTCACTAGGCAGTG |  |
| Bs-85-170-F | GTACATATGAATGAGCAGATTCCACATGAC |  |
| Bs-85-F | CGCTGTTTGGTGTTAATGCGATT**CAC**GGAATGGATGGCAGCGCGCATA |  |
| Bs-85-R | GGATATGCGCGCTGCCATCCATTCC**GTG**AATCGCATTAACACCAAAC |  |
| Bs-170-F | GAGAGCGGATGACTTCATTGACATG**ATC**GACGCGTTCGGTGCTGTGG |  |
| Bs-170-R | GTCCCACAGCACCGAACGCGTC**GAT**CATGTCAATGAAGTCATCCGC |  |
| Bs-85-170-R | GTACTCGAGTTAACTTTTTCGTCTGATTCCGCTC |  |

The bold nucleotides denote the mutated codons. The recognition sequence of *Nde*I and *Xho*I are underlined.
